# Genome-wide association analyses identified novel susceptibility loci for pulmonary embolism among Han Chinese population

**DOI:** 10.1186/s12916-023-02844-4

**Published:** 2023-04-19

**Authors:** Zhu Zhang, Haobo Li, Haoyi Weng, Geyu Zhou, Hong Chen, Guoru Yang, Ping Zhang, Xiangyan Zhang, Yingqun Ji, Kejing Ying, Bo Liu, Qixia Xu, Yongjun Tang, Guangfa Zhu, Zhihong Liu, Shuyue Xia, Xiaohong Yang, Lixia Dong, Ling Zhu, Mian Zeng, Yadong Yuan, Yuanhua Yang, Nuofu Zhang, Xiaomao Xu, Wenyi Pang, Meng Zhang, Yu Zhang, Kaiyuan Zhen, Dingyi Wang, Jieping Lei, Sinan Wu, Shi Shu, Yunxia Zhang, Shuai Zhang, Qian Gao, Qiang Huang, Chao Deng, Xi Fu, Gang Chen, Wenxin Duan, Jun Wan, Wanmu Xie, Peng Zhang, Shengfeng Wang, Peiran Yang, Xianbo Zuo, Zhenguo Zhai, Chen Wang, on behalf of the China pUlmonary Thromboembolism REgistry Study (CURES) investigators

**Affiliations:** 1 Department of Pulmonary and Critical Care Medicine, China-Japan Friendship Hospital; National Center for Respiratory Medicine; Institute of Respiratory Medicine, Chinese Academy of Medical Sciences; National Clinical Research Center for Respiratory Diseases, Beijing, 100029 China; 2 China-Japan Friendship Hospital, Chinese Academy of Medical Sciences & Peking Union Medical College; Department of Pulmonary and Critical Care Medicine, Center of Respiratory Medicine, China-Japan Friendship Hospital; National Center for Respiratory Medicine; Institute of Respiratory Medicine, Chinese Academy of Medical Sciences; National Clinical Research Center for Respiratory Diseases, Beijing, 100029 China; 3 Shenzhen WeGene Clinical Laboratory; WeGene, Shenzhen Zaozhidao Technology Co. Ltd; Hunan Provincial Key Lab On Bioinformatics, School of Computer Science and Engineering, Central South University, Shenzhen, 518042 China; 4 Department of Bioinformatics and Biostatistics, SJTU-Yale Joint Center for Biostatistics, College of Life Science and Biotechnology, Shanghai Jiao Tong University, Shanghai, 200240 China; 5 Department of Pulmonary and Critical Care Medicine, the First Affiliated Hospital of Chongqing Medical University, Chongqing, 400016 China; 6 Department of Pulmonary and Critical Care Medicine, Weifang No.2 People’s Hospital, Weifang, 261021 China; 7 Department of Pulmonary and Critical Care Medicine, Dongguan People’s Hospital, Dongguan, 523059 China; 8 Department of Pulmonary and Critical Care Medicine, Guizhou Provincial People’s Hospital, Guiyang, 550002 China; 9 Department of Pulmonary and Critical Care Medicine, Shanghai East Hospital Affiliated by Tongji University, Shanghai, 200120 China; 10 Department of Respiratory Medicine, Sir Run Run Shaw Hospital, Zhejiang University School of Medicine, Hangzhou, 310020 China; 11 Department of Pulmonary and Critical Care Medicine, Department of Clinical Microbiology, Zibo City Key Laboratory of Respiratory Infection and Clinical Microbiology, Linzi District People’s Hospital, Zibo, 255400 China; 12 Department of Pulmonary and Critical Care Medicine, the First Affiliated Hospital of University of Science and Technology of China, Hefei, 230001 China; 13 Department of Pulmonary and Critical Care Medicine, Xiangya Hospital Central South University, Changsha, 410008 China; 14 Department of Pulmonary and Critical Care, Beijing Anzhen Hospital, Capital Medical University, Beijing, 100029 China; 15 Fuwai Hospital, Chinese Academy of Medical Science; National Center for Cardiovascular Diseases, Beijing, 100037 China; 16 Department of Pulmonary and Critical Care Medicine, Central Hospital Affiliated to Shenyang Medical College, Shenyang, 110001 China; 17 Department of Pulmonary and Critical Care Medicine, People’s Hospital of Xinjiang Uygur Autonomous Region, Xinjiang, 830001 China; 18 Department of Pulmonary and Critical Care Medicine, Tianjin Medical University General Hospital, Tianjin, 300050 China; 19 Department of Pulmonary and Critical Care Medicine, Shandong Provincial Hospital, Jinan, 250021 China; 20 Department of Medical Intensive Care Unit, The First Affiliated Hospital, Sun Yat-Sen University, Guangzhou, 510080 China; 21 Department of Pulmonary and Critical Care Medicine, The Second Hospital of Hebei Medical University, Shijiazhuang, 050004 China; 22 Department of Pulmonary and Critical Care Medicine, Beijing Chao-Yang Hospital, Capital Medical University, Beijing, 100026 China; 23 State Key Laboratory of Respiratory Disease and National Clinical Research Center for Respiratory Disease, the First Affiliated Hospital of Guangzhou Medical University, Guangzhou Medical University, Guangzhou, 510230 China; 24 Department of Pulmonary and Critical Care Medicine, Beijing Hospital, Beijing, 100080 China; 25 Department of Pulmonary and Critical Care Medicine, Beijing Jishuitan Hospital, Beijing, 100035 China; 26 China-Japan Friendship Hospital, Capital Medical University; Department of Pulmonary and Critical Care Medicine, Center of Respiratory Medicine, China-Japan Friendship Hospital; National Center for Respiratory Medicine; Institute of Respiratory Medicine, Chinese Academy of Medical Sciences; National Clinical Research Center for Respiratory Diseases, Beijing, 100029 China; 27 Institute of Clinical Medical Sciences, China-Japan Friendship Hospital; Department of Pulmonary and Critical Care Medicine, Center of Respiratory Medicine, China-Japan Friendship Hospital; National Center for Respiratory Medicine; Institute of Respiratory Medicine, Chinese Academy of Medical Sciences; National Clinical Research Center for Respiratory Diseases, Beijing, 100029 China; 28 Institute of Clinical Medical Sciences, China-Japan Friendship Hospital; National Center for Respiratory Medicine; Institute of Respiratory Medicine, Chinese Academy of Medical Sciences; National Clinical Research Center for Respiratory Diseases, Beijing, China 100029; 29 Institute of Basic Medical Sciences, Chinese Academy of Medical Sciences, Peking Union Medical College, Beijing, 100005 China; 30 Beijing Pediatric Research Institute, Beijing Children’s Hospital, Capital Medical University, National Center for Children’s Health, Beijing, 100045 China; 31 Department of Epidemiology and Biostatistics, School of Public Health, Peking University, Beijing, 100191 China; 32 Department of Dermatology, China-Japan Friendship Hospital, Beijing, China; Department of Pharmacy, China-Japan Friendship Hospital, No. 2, East Yinghua Road, Chaoyang District, Beijing, 100029 China; 33 Department of Pulmonary and Critical Care Medicine, Center of Respiratory Medicine, China-Japan Friendship Hospital, Beijing, China; 34 National Center for Respiratory Medicine, Beijing, China; 35 Institute of Respiratory Medicine, Chinese Academy of Medical Sciences, Beijing, China; 36 National Clinical Research Center for Respiratory Diseases, Beijing, China; 37 Chinese Academy of Medical Sciences, Peking Union Medical College, Beijing, China; 38 Department of Respiratory Medicine, Capital Medical University, Beijing, China

**Keywords:** Pulmonary embolism, GWAS, Han Chinese

## Abstract

**Background:**

A large proportion of pulmonary embolism (PE) heritability remains unexplained, particularly among the East Asian (EAS) population. Our study aims to expand the genetic architecture of PE and reveal more genetic determinants in Han Chinese.

**Methods:**

We conducted the first genome-wide association study (GWAS) of PE in Han Chinese, then performed the GWAS meta-analysis based on the discovery and replication stages. To validate the effect of the risk allele, qPCR and Western blotting experiments were used to investigate possible changes in gene expression. Mendelian randomization (MR) analysis was employed to implicate pathogenic mechanisms, and a polygenic risk score (PRS) for PE risk prediction was generated.

**Results:**

After meta-analysis of the discovery dataset (622 cases, 8853 controls) and replication dataset (646 cases, 8810 controls), GWAS identified 3 independent loci associated with PE, including the reported loci *FGG* rs2066865 (*p*-value = 3.81 × 10^−14^), *ABO* rs582094 (*p*-value = 1.16 × 10^−10^) and newly reported locus *FABP2* rs1799883 (*p*-value = 7.59 × 10^−17^). Previously reported 10 variants were successfully replicated in our cohort. Functional experiments confirmed that *FABP2-A163G*(rs1799883) promoted the transcription and protein expression of *FABP2*. Meanwhile, MR analysis revealed that high LDL-C and TC levels were associated with an increased risk of PE. Individuals with the top 10% of PRS had over a fivefold increased risk for PE compared to the general population.

**Conclusions:**

We identified *FABP2*, related to the transport of long-chain fatty acids, contributing to the risk of PE and provided more evidence for the essential role of metabolic pathways in PE development.

**Supplementary Information:**

The online version contains supplementary material available at 10.1186/s12916-023-02844-4.

## Background

Pulmonary embolism (PE) is a complex and multifactorial disease, together with deep vein thrombosis (DVT), commonly referred as venous thromboembolism (VTE). Twin studies have estimated the heritability of VTE to be approximately 50%, indicating that genetic factors may play a significant role in the pathogenesis of the disease [1]. Over the past decades, family and population studies have discovered dozens of variants across the genome that contribute to the genetic risk of PE or VTE [2, 3]. The largest meta-analysis of genome-wide association study (GWAS) for VTE has identified 34 independent genetic signals [4]. Most of the reported associated loci regulate the coagulation and anticoagulation functions [4, 5], which are vital disease-causing mechanisms in PE. Additionally, platelet, inflammation and erythrocytes have also been associated with the risk [4]. However, we still have limited knowledge of the genetic architecture of PE, leaving a large proportion of heritability unexplained [6, 7].

The global disease burden of PE has been steadily increasing in the past decade, affecting 100–200 per 100,000 individuals in western countries. Nevertheless, the prevalence of PE in EAS is 1/3–1/5 of that in EUR [8]. Little is known about the genetic and other factors accounting for PE prevalence between East Asians (EAS) and European ancestry (EUR). Studies have suggested that ancestry-specific allele frequencies may explain the differences [9]. Genetic studies with diverse populations are valuable for identifying more genetic risk factors of PE and maximizing the relevance of findings across populations [10]. However, there have been few genetic studies for PE among EAS.

Genetic studies of VTE have been performed in subjects with EUR or African American (AA) ancestry. However, due to varying minor allele frequencies (MAF) across different populations, some of the associated variants identified in one population may not be replicated in another population. For example, rs6025 in *F5* (Factor V Leiden, FVL), the well-known leading single nucleotide polymorphism (SNP) in EUR VTE patients, has been rarely reported among EAS, with a MAF reported to be 0.024 in EUR and 0 in EAS [11–13]. Similarly, variants in *THBD* have been reported to be associated with VTE in AA but not in EUR [14–16]. Such inconsistency makes it unreliable to generalize the genetic findings from EUR to other populations. Direct application of the PE risk assessment models with genetic factors discovered in the EUR may lead to inaccurate estimates of the actual PE risk among EAS, exacerbating health disparities in diverse populations [17, 18].

Till now, there is no solid evidence of the PE risk assessment base on genetic study in EAS. To accelerate our understanding of the genetic basis for PE in EAS, we performed a large-scale genome-wide association study in the Han Chinese population and developed a population-specific polygenic risk score (PRS) to identify sub-populations at higher risk of PE.

## Methods

### Study design and participants

DNA samples and phenotype data were collected from a Han descent population (Additional File 1: Fig. S1). All samples were collected from the China Pulmonary Thromboembolism Registry Study (CURES), recruited from 2016 to 2020 [19]. We identified patients with our inclusion criteria, while ancestry-matched controls were obtained in collaboration with WeGene [20].

This study was conducted under the human and ethical research principles of The Ministry of Science and Technology, People’s Republic of China (Regulation of the Administration of Human Genetic Resources, July 1, 2019). All the participants provided informed consent and agreed to participate in this research under a protocol approved by the Ethical Committee of China-Japan Friendship Hospital (Cases) and WeGene (Controls). The study’s ethical approval was obtained from the Ethics Committee in China-Japan Friendship Hospital (2016-SSW-7).

### Genotyping and genome-wide quality-control procedures

DNA of cases and controls were extracted from whole blood or saliva samples. All participants were genotyped at WeGene Lab using a customized Illumina WeGene V2 Array by Illumina iScan System, which contained roughly 700,000 markers covering the whole genome. The customized array was originally designed from the Infinium Global Screening Array BeadChip, which specifically included around 560,000 genome-wide backbone markers for optimized genome-wide association studies in the Chinese population and other markers for clinical research and quality control. Genotype imputation was conducted using ChinaMAP (http://www.mbiobank.com/login/?next=%2Fimputation%2F), an online imputation server for East Asian population genotype imputation [21, 22]. Genotype calling was performed using Illumina GenomeStudio software. Quality control was performed before further analyses. Individuals were excluded based on gender mismatches, disproportionate levels of individual missingness (> 0.05), evidence of relatedness (removing one from each pair within 2nd-degree identified by KING [23], inbreeding coefficient above 0.2 or below − 0.2), and being of non-Han Chinese. The patients of discovery dataset were recruited from 2016 to 2018, while the patients of replication dataset were recruited from 2018 to 2020. The top ten principal components (PCs) were calculated using GCTA [24].

### Genome-wide association testing, and meta-analysis

For each phase, all genotyped variants passing quality control on autosomal chromosomes were tested for association with PE through logistic regression adjusting for age, sex, and top ten principal components (PCs) using PLINK [25]. The genome-wide significance threshold was set at *p*-value < 5 × 10^−8^. Association summary statistics were combined for variants common to discovery stage and replication stage, and then for variants common to all two phases, in fixed effects models using METAL [26]. Cochran’s Q statistic was used to test for heterogeneity and the *I*^2^ statistic was used to quantify variation due to heterogeneity. To visualize the results, a Manhattan plot and a quantile–quantile (Q-Q) plot were generated using the R package “qqman.” A regional association plot for the genomic region within 500 Kb of the top hit was generated using LocusZoom software [27], and a forest plot for the most significant SNP association was generated using revman.

### Gene-based testing analysis

Gene-based testing was performed using FUMA [28, 29] software. Input SNPs were mapped to 15,756 protein-coding genes, so we set 3.17 × 10^−6^ as the Bonferroni-corrected significance threshold (0.05/15756 = 3.173 × 10^−6^). The python package *assocplots* was used to produce Manhattan plots and QQ-plots [30].

### Cellular experiments

#### Cells and reagents

The HEK293T cells (ATCC, CRL-3216, LabWecom, China) were maintained in DMEM (11,965,092, Gibco, America) supplemented with 10% FBS (10099141C, Gibco, America) and Penicillin–Streptomycin (15,140,163, Gibco, America) with indicated proportion.

#### Plasmids and antibodies

The GFP-Tagged full-length target gene (Wild Type, WT) and its mutant type (MT) were constructed into eukaryotic expression PEGFP-N1, respectively. Recombinant PEGFP-N1s encoding the target gene and its MT were constructed by PCR-based amplification of cDNA from the sequence. The WT and MT cDNA of the target gene were subcloned into the eukaryotic expression PEGFP-N1, respectively. The antibody to GFP (AE012, human-specific) was purchased from Abclonal.

#### Plasmid transfection and immunofluorescence assay

HEK293T cells were cultured and then seeded in 6-well plates and the cell density was 350,000 per well. After overnight incubation, cells were transfected with PEGFP-N1, target gene-WT and target gene-MT, respectively. Plasmids were pre-incubated with Opti-MEM™ (31,985,070, GIbco, America) and jetPRIME® Transfection Reagent (114–15, Polyplus, France) as instructed on the manual. After 72 h, fluorescence microscopy were used to assess the transfection efficiency.

#### Western blot analysis

Western blotting was performed as described previously [31]. Briefly, cells were lysed with lysis buffer (1% TritonX 100, 20 mM Tris–HCl pH 8.0, 250 mM NaCl, 3 mM EDTA pH 8.0), 3 mM EGTA (pH 8.0) with the pH adjusted to 7.6, and complete protease inhibitor cocktail (CW2200, CWBIO, China) on ice for 30 min. Lysates were eluted by boiling 10 min with 2 Χ sample buffer (100 mM Tris–HCl, pH 6.8, 2% SDS, 10% glycerol, 0.1% bromophenol blue, 1% β-mercaptoethanol) and were separated by 10% SDS/PAGE, followed by examination of expression levels of the indicated proteins. β-Tubulin served as an internal control.

#### Quantitative PCR analysis

Gene expression was analyzed by three-step q-RT–PCR (qPCR). Total RNA were extracted from HEK293T cells transfected plasmids using TRIzol reagent (T9424, Sigma, Germany). Following the manufacturer’s instructions, RNA were reverse-transcribed in a 20 μl reaction volume (42 °C, 30 min; 95 °C, 5 min) using a QuantiTect Reverse Transcription Kit (KR118, TIANGEN, China). cDNA was then amplified using a SYBR Green I Master mix (FP205, TIANGEN, China) and the ABI 7500 Fast Real Time PCR system (ABI, America). All tests were carried out on duplicate reaction mixtures in 96-well plates. The relative expression of the gene of interest was determined using the 2^–ΔΔCt^ method, with GAPDH as the internal control.

### Mendelian randomization(MR) analysis

In order to detect the cause relationship between PE and lipoprotein associated triat, we performed two-sample Mendelian randomization (MR) analysis. Summary statistics for exposure were obtained from Biobank Japan Project (http://jenger.riken.jp/en/), which contained individuals of similar EAS ancestry as Chinese population to avoid population stratification. We estimated the causal effect of exposure on outcome using two‐sample MR method. The inverse variance weighted (IVW) method [32] was used in the main MR analyses, and the maximum likelihood weighted median and penalized weighted median [33] approaches were employed as sensitivity analysis. MR-Egger [34] method was used to detect the directional pleiotropy according to the intercept of weighted linear regression of the SNP‐outcome coefficients on SNP‐exposure coefficients. Results were considered statistically significant at *p*-value < 0.017 (0.05/3). The MR analysis was performed using the TwoSampleMR [35] package and MR-PRESSO [33] package in R software (version 3.4).

### Heritability and LD-score regression

LDSC v.1.0.1 was used to calculate the heritability on the liability scale [36].

### Polygenic risk score analysis

We derived the PRS from PE associated variants in the Han Chinese population. The PRS training cohort including cases and controls of discovery stage, and the PRS testing cohort including cases and controls of replication stage. PRSs of the test cohort were calculated using the Polygenic Risk Score software (PRSice-2) [37], based on the summary statistics of the training group. The performance of a series of cutoff of PE association p-values for selection of SNP markers was assessed by the Area Under the Curve (AUC) for ROC. The p-value cutoff with the largest AUC was adopted. According to the PRS, individuals were divided into seven intervals from low to high (< 10th, 10–20th, 20–30th, 30–70th, 70–80th, 80–90th, < 90th), and the odds ratio of each interval relative to the baseline data (30–70th) is calculated. Finally, we used PLINK to calculate the performance of other PRS available in the literature in the testing cohort and compared them with our PRS.

## Results

### GWAS and replication analysis

**Fig. 1:**
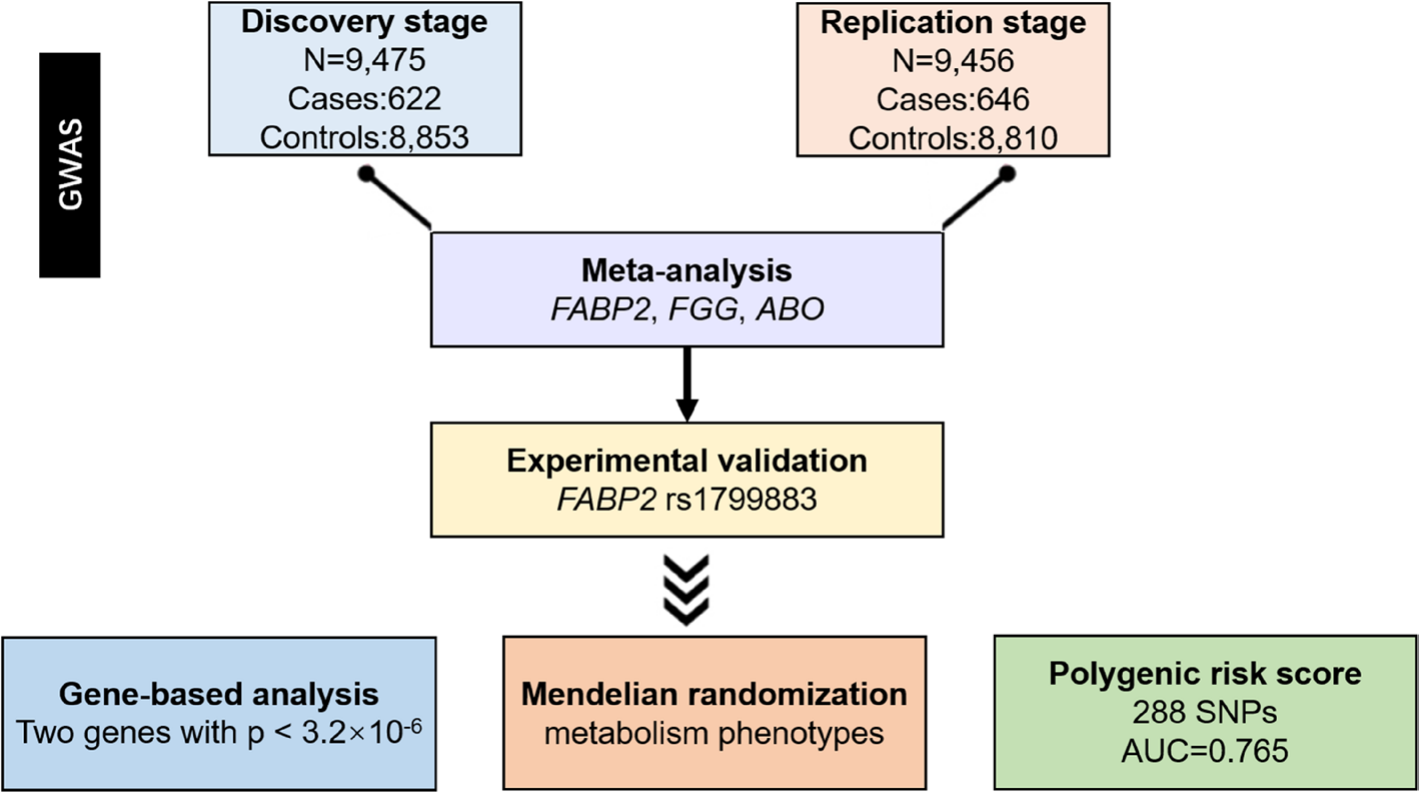
Study workflow

In the discovery stage, we consecutively recruited 624 cases (Fig. 1). 622 PE cases passed QC and were analyzed, of which the mean (+ / − SD) age was 62 (+ / − 19) years old, and 323 (52%) were male. A total number of 8853 controls were collected. Although there are statistically significant differences (*p*-value < 0.05) in age and sex between cases and controls, the association results are unlikely to be biased as the susceptible variants are generally independent regardless of age and sex.

**Fig. 2:**
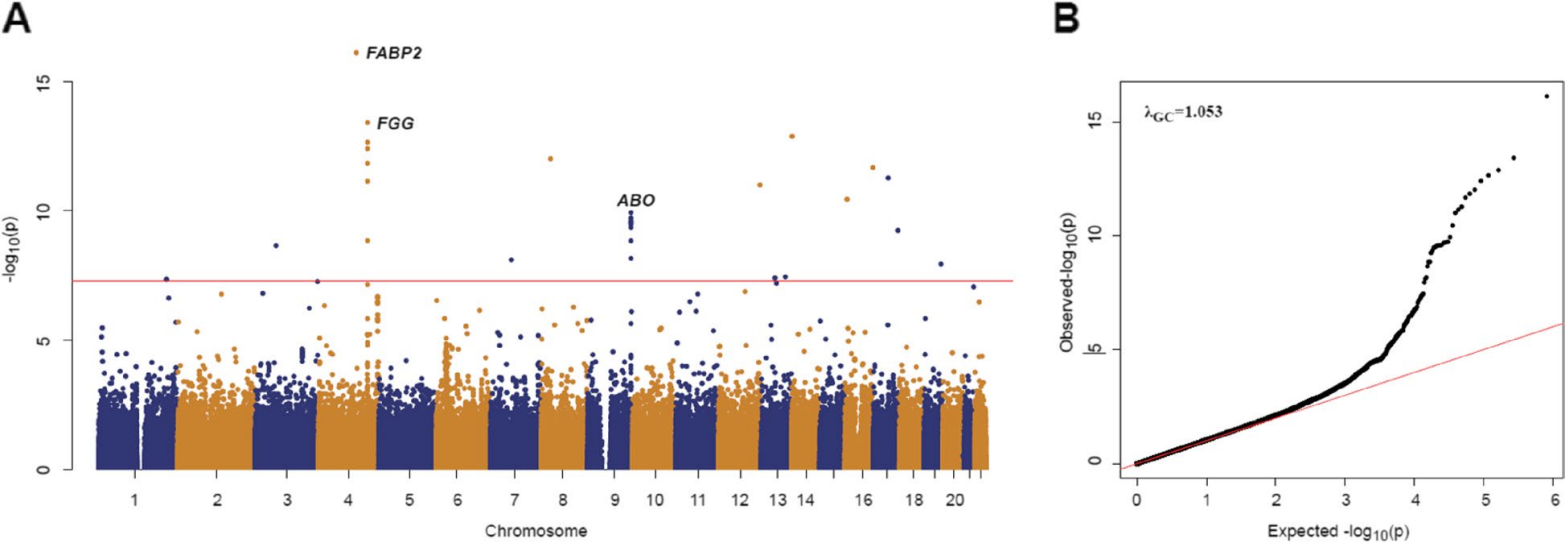
Manhattan plot and QQ plot of GWAS meta-analysis. **A** Manhattan plot of the results from meta-analysis. The *y*-axis represents –log_10_(*p*-values) for the association of variants with VTE using a logistic regression model. The horizontal line represents the threshold for genome-wide significance. Representative loci with genome-wide significance are labeled. **B** QQ plot of the results from meta-analysis. *λ*_*GC*_ denotes the genomic inflation factor. The *x*- and *y*-axes represent expected and observed − log_10_(*p*-values), respectively

**Fig. 3:**
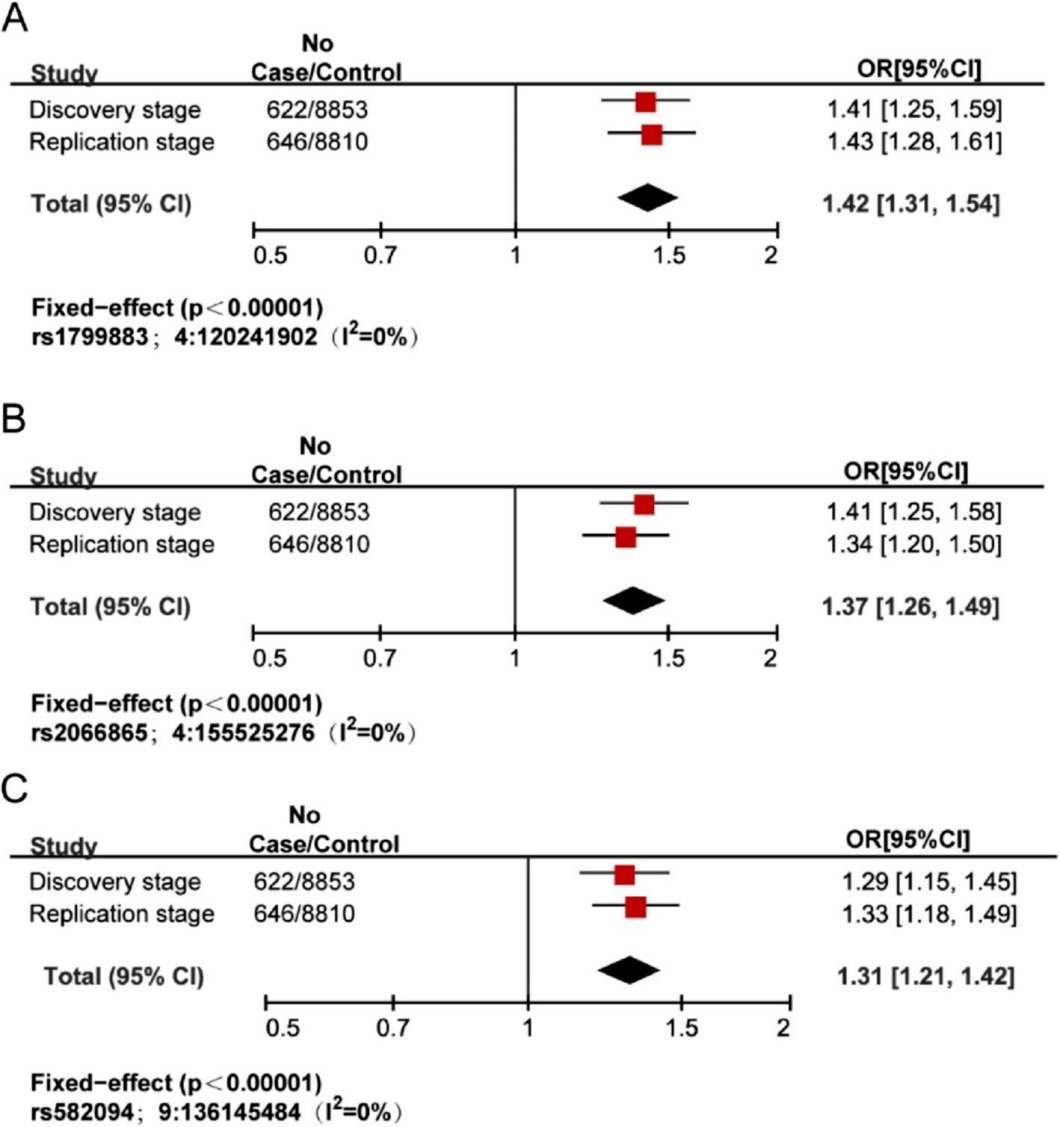
Forest plots for 3 loci associated with pulmonary embolism. Study cohorts, sample sizes (case and control), and estimated odds ratios (OR) for **A** rs1799883, **B** rs2066865, and **C** rs582094.The vertical line corresponds to the null hypothesis (OR = 1). The horizontal lines and square brackets indicate 95% confidence intervals (95% CI). Areas of the boxes are proportional to the weight of the study. Diamonds represent combined estimates for fixed‐effect analysis. The heterogeneity index, *I*^2^ (0–1) was also measured which quantifies the proportion of the total variation due to heterogeneity. All statistical tests were two-sided and no adjustments were made for multiple comparisons

In replication stage, 647 acute PE cases and 8820 controls were included as additional independent samples. Six hundred forty-six PE cases and 8810 controls passed QC and were analyzed. The mean (+ / − SD) age was 62 (+ / − 15) years old, and 341 (53%) were male in the case group. The characteristics of the participants were presented in Table 1. Only *FABP2* rs1799883 and *FGG* rs2066865 reached genome-wide significance in the discovery stage. We then performed the GWAS meta-analysis based on the discovery and replication stage. Finally, we identified 16 genome-wide significant variants in the meta-analysis, where *FGG* rs2066865 (*p*-value = 3.81 × 10^−14^) and *ABO* rs582094 (*p*-value = 1.16 × 10^−10^) were known loci in the coagulation pathway, and *FABP2* rs1799883 (*p*-value = 7.59 × 10^−17^) was newly reported (Figs. 2 and 3). The three variants were independent of each other and the leading SNPs are in strong linkage disequilibrium with considerable imputed variants of similar statistical associations (Table 2, Additional File 1: Fig. S1), the remaining 13 loci were neither genome-wide significant in the discovery stage nor previously known to be associated with VTE (Additional File 2: Table S1, Additional File 2: Table S2). PCA (Additional File 1: Fig. S2) and QQ plot (Fig. 2) showed no population stratification and inflation of test statistics (λ_GC_ = 1.053).

**Table 1:** Demographic, clinical characteristics, and risk factors of the patients included

Characteristics	Discovery stage		Replication stage	
	Case	Control	Case	Control
	*N* = 622	*N* = 8853	*N* = 646	*N* = 8810
**Demographic characteristics**				
Age, year, mean (SD)	62 (19)	59 (9)	62 (15)	59 (8)
Male, *n* (%)	323 (52)	4041 (46)	341 (53)	4041 (46)
BMI, kg/m^2^, mean (SD)	24.4 (3.9)	-	24.5 (3.6)	-
PE alone,* n* (%)	331 (53)	-	340 (53)	-
PE combined DVT, *n* (%)	291 (47)	-	306 (47)	-
**Complications and risk factors**		-		-
Cardiovascular disease, *n* (%)	311 (50)	-	311 (48)	-
Chronic pulmonary disease, *n* (%)	105 (17)	-	118 (18)	-
Cancer, *n* (%)	60 (10)	-	62 (10)	-
Metabolic and endocrine diseases, *n* (%)	108 (17)	-	101 (16)	-
Smoking history, *n* (%)	198 (32)	-	182 (28)	-
	*N* = 218		*N* = 220	
**Thrombophilia test**		-		-
Plasma antithrombin activity < 70%, *n*/*N* (%)	32 (15)	-	32 (15)	-
Plasma protein S activity < 60%, *n*/*N* (%)	36 (17)	-	42 (19)	-
Plasma protein C activity < 70%, *n*/*N*, *n*/*N* (%)	42 (19)	-	49 (22)	-

*BMI* Body mass index, *PE* Pulmonary embolism, *SD* Standard deviation

**Table 2:** Independent genome-wide significant lead SNPs verified to be associated with PE

Chromosome position	rsID	EA/OA	Nearest gene	Discovery stage			Replication stage			Meta		
				EAF (case/control)	OR (95% CI)	*P*-value	EAF (case/control)	OR (95% CI)	*P*-value	OR (95% CI)	*P*-value	*I*^2^
4:120,241,902	rs1799883	T/C	*FABP2*	0.31 (0.39/0.31)	1.41 (1.25–1.59)	1.05 × 10^−8^	0.31 (0.39/0.30)	1.43 (1.28–1.61)	1.25 × 10^−9^	1.42 (1.31–1.54)	7.59 × 10^−17^	0
4:155,525,276	rs2066865	A/G	*FGG*	0.51 (0.57/0.52)	1.41 (1.25–1.58)	9.85 × 10^−9^	0.52 (0.55/0.52)	1.34 (1.20–1.50)	6.19 × 10^−7^	1.37 (1.26–1.49)	3.81 × 10^−14^	0
9:136,145,484	rs582094	T/A	*ABO*	0.43 (0.50/0.43)	1.29 (1.15–1.45)	2.25 × 10^−5^	0.44 (0.49/0.43)	1.33 (1.19–1.49)	1.18 × 10^−6^	1.31 (1.21–1.42)	1.16 × 10^−10^	0

Chromosome position: based on hg19/GRCh37 build. *rsID* rs number, *EA* Effect allele, *OA* Other allele, *EAF* Effect allele frequency, *OR* Odds ratio, *CI* confidence interval

### Replication of associations peviously identified loci in EAS population

We compiled VTE susceptibility SNPs from the pevious GWAS literature and assessed the associations of 22 previously reported VTE associated variants in our Han Chinese samples [3, 16, 38–46]. For 10 out of 22 variants, the association signals were successfully replicated at the Bonferroni-corrected significance level of 2.27 × 10^−3^ (0.05/22). The most significant SNP was rs505922 near *ABO*, with *p*-value of 3.81 × 10^−14^ and odds ratio (OR, 95% confidence interval [CI]) of 1.31 (1.20–1.42). Nine additional SNPs, at five gene, also reached the threshold for significance. These were rs657152*,* rs630014 and rs687289 near *ABO*, rs6825454 and rs2070011 near *FGA*, rs2066865 and rs6536024 near *FGG*, rs4253399 near *F11*, and rs13084580 near *CSRNP1*. Other previously reported candidate genes (*F2*, *F5*, *PROS1*, *PROCR*, *NME7*, *SLC44A2*, *THBD*, *SMAP1/B3GAT2*, *PEPD*, *GP6*) for recurrent VTE showed no or very weak association in our cohort (Additional File 2: Table S3).

### Gene-based analysis

We further performed a gene-based analysis using functional mapping and annotation (FUMA) MAGMA to prioritize candidate genes associated with PE. After aggregating the association signal of all SNPs in each gene, we identified two genes (*FABP2* and *FGG*) reaching the Bonferroni-corrected threshold (*p*-value < 3.17 × 10^−6^), and *FGG* was previously known to be associated with PE (*p*-value = 1.45 × 10^−6^) (Additional File 1: Fig. S3, Additional File 2: Table S4). However, we noted that *ABO* is annotated as a “processed transcript” in the database and, therefore, it was not incorporated into this analysis.

### FABP2-A163G promotes gene transcription and protein expression of FABP2

**Fig. 4:**
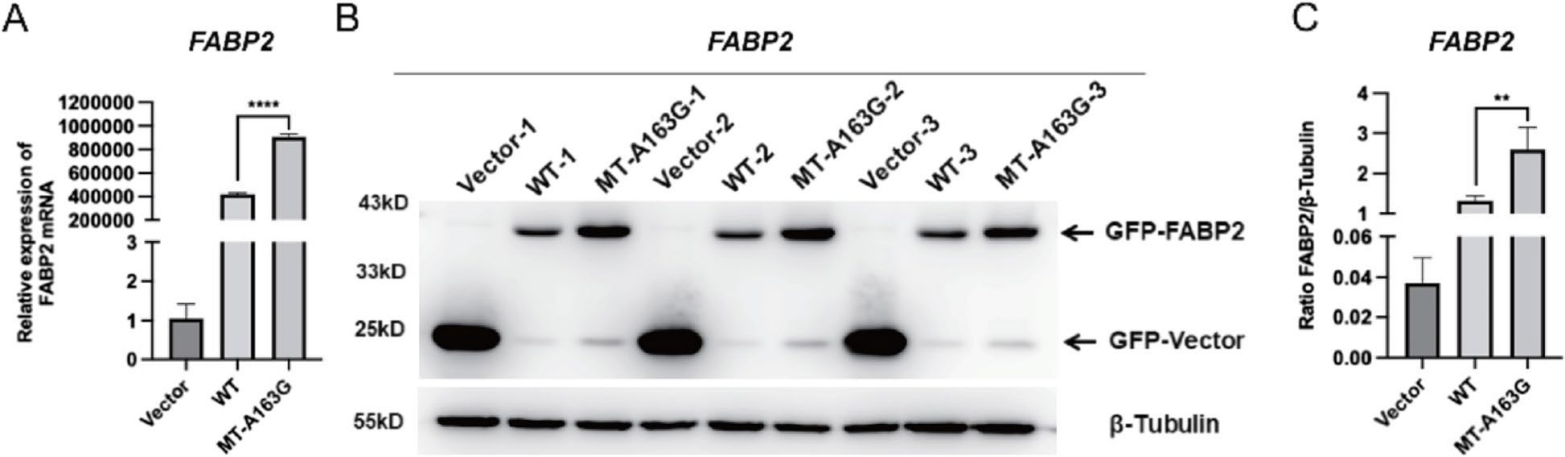
*FABP2* mutant enhanced itself expression compared to WT by qPCR and Western-blot experiments.** A** qPCR results showed effects on transcription level of *FABP2* by transfected Vector, WT and MT-A163G plasmids. **B**, **C** Western Blots results showed effects on protein expression level of *FABP2* by transfected vector, WT, MT-A163G plasmids. WT, wildtype; MT-A163G, mutation type with *FABP2*; *P < 0.05; **P < 0.01; ***P < 0.001; ****P < 0.0001; ns, not significant

To explore the influence of the variant on *FABP2* expression, PEGFP-N1, FABP2-WT, FABP2-A163G plasmids were transfected into HEK293T cells, and the qPCR results suggested that FABP2-A163G promoted FABP2 transcription compared to FABP2-WT (Fig. 4A, Additional File 1: Fig. S4).

To further examine the effect on FABP2 protein expression, the whole cell lysate from HEK293T cells transfected with EGFP-N1, FABP2-WT, and FABP2-A163G plasmids were harvested. Western blotting results suggested that FABP2-A163G markedly promoted FABP2 protein expression compared to FABP2-WT (Fig. 4B, C).

### Mendelian randomization (MR) of metabolism phenotypes and PE

Our GWAS analysis has implicated a strong relationship between *FABP2* polymorphism and PE. *FABP2* polymorphism is known to be significantly associated with serum total cholesterol and LDL-C [47], which is also proven by our data (Additional File 1: Fig. S5). Previous studies [4, 38] have implied that lipid metabolic traits may be involved in PE pathogenesis. To further investigate the potential causal relationship, we conducted MR analysis of PE and 3 metabolic related phenotypes: total cholesterol (TC), triglycerides (TG), and low-density lipoprotein-cholesterol (LDL-C). The results indicated that high TC, and LDL-C levels were associated with increased risk of PE while TG was not causally related with the risk of PE (TC: OR = 1.42; 95% CI = 1.24–1.61; *p*-value < 0. 001; LDL-C: OR = 1.21; 95% CI = 1.13–1.29; *p*-value < 0. 001; TG: *p*-value = 0.357). Results from maximal likelihood and MR-PRESSO analysis were consistent with IVW (Additional File 1: Fig. S6).

### Heritability and LD-score regression

Using LDSC and common variants outside of the HLA region, we found that the estimated heritability of PE was comparable between EAS and EUR (0.16 ± 0.03). The LD score regression generated an intercept of 1.01 ± 0.01 with a *p*-value = 0.152.

### PRS analysis

**Fig. 5:**
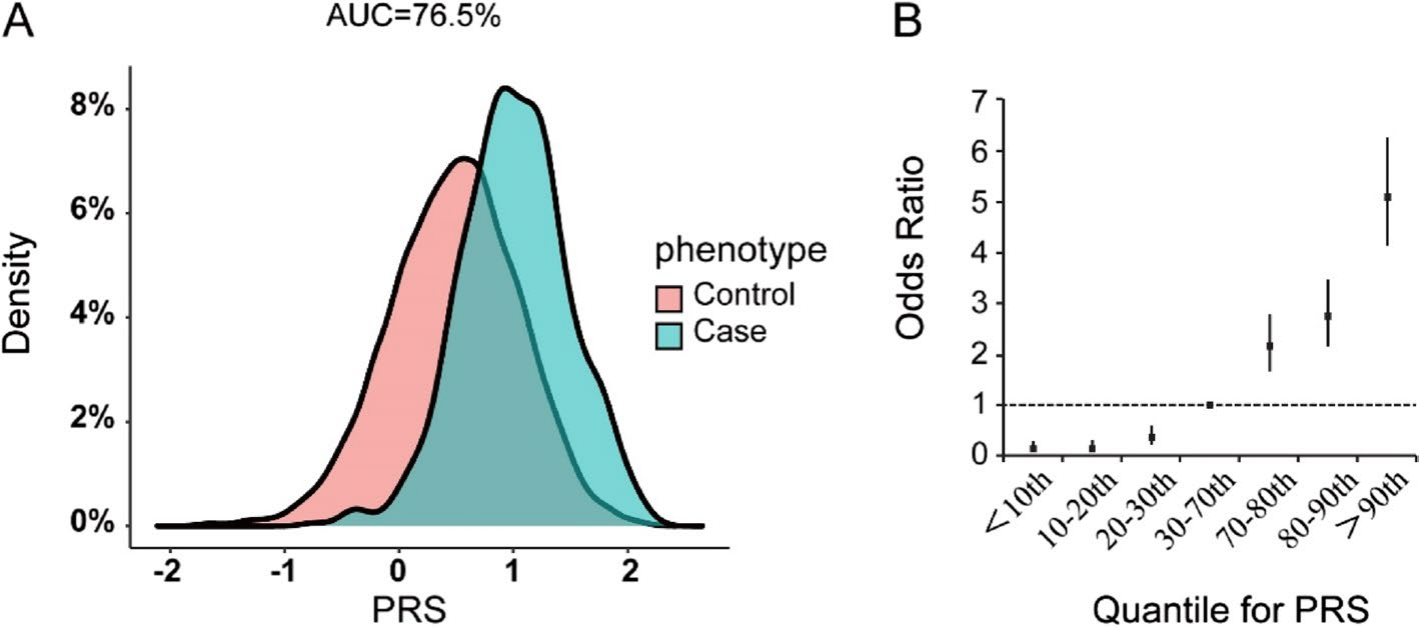
Ancestry-specific polygenic risk score (PRS) distribution and odds ratio of PRS quantiles. **A** Density distribution of the standardized PRS in cases and controls. AUC, area under the receiver operating characteristic curve. **B** Odds ratio of samples divided into different quantile bins using PRS with the 30–70% quantile chosen as the reference. Error bars indicate the 95% confidence interval

We generated a 288-variant (Additional File 2: Table S5) PRS under a penalized regression framework using discoverage stage as the training set and replication stage as the testing set. The receiver operating characteristic curve (ROC) in the testing set achieved an area under the curve (AUC) of 0.765 (Additional File 1: Fig. S7). The distributions of standardized PRS for cases and controls in the testing data were illustrated in Fig. 5A. Individuals in the top 10% group of PRS had a 5.08-fold of PE risk relative to the general population (30th–70th quantile) (Fig. 5B, Additional File 2: Table S6). Our 288-variant PRS had a better performance than three publicly available genome-wide PRSs in the testing set (Additional File 1: Fig. S8).

## Discussion

As most large-scale genetic studies of VTE have been conducted in EUR ancestry [4, 45, 48, 49], we performed the first GWAS in the Han Chinese population to expand the genetic landscape of PE. We identified three genome-wide significant loci, of which two were known to be associated with PE (*FGG*, *ABO*). Another locus at *FABP2* reached the significance threshold both in the discovery stage and meta-analysis. The risk allele at the *FABP2* locus (rs1799883) is reported as a functional variant to increase the gene expression by qPCR and Western-blot expreriments. The results showed that the carriers of that mutation have higher blood TC and LDL-C [50]. We further performed MR analysis and found that increased levels of LDC-C and TC were associated with a higher risk of PE, which implied the inhibition of LDL-C and TC to be a potential measure of PE prevention.

Some significant loci for the risk of VTE have been reported in European ancestry in the past but have not been validated in the Asian population, we attempted to replicate the previously known loci identified among the EUR population in the current study. In our study, 10 of the 22 leading variants were replicated, located on *FGG*, *F11*, *ABO*, *CSRNP1*, and *FGA*. The lack of replication in the current study might be partially attributed to insufficient power and diverse LD patterns across ancestries rather than different biological effects. These findings further emphasize the importance of including diverse ancestral groups in genomic studies to maximize the power for detecting disease associations.

Fatty acid binding proteins (FABPs) are key proteins in lipid transport. FABP2 can traffic lipids from the intestinal lumen to enterocytes and bind superfluous fatty acids to maintain a steady pool of fatty acids in the epithelium. *FABP2* polymorphism is known to be significantly associated with serum total cholesterol and LDL-C [47]. Based on the above evidence, we speculate that variants in *FABP2* may contribute to PE through lipid metabolism function. Our findings were consistent with previous studies on the role of metabolic traits in PE [51], which is the first time to be verified among the EAS population. Lipid-lowering drugs for prevention or even adjunctive therapy of PE have been proposed in many clinical trials [52]. For example, statins contribute to PE prevention through anti-inflammatory and LDL-lowering effects [53]. Proprotein convertase subtilisin/kexin type (PCSK9) inhibitor has also been identified to lower the risk of VTE by LDL reduction [54]. Our study provided a shred of robust evidence that lipid-lowering therapy may also be considered to prevent PE occurrence in the EAS population.

In addition to *FABP2*, *FGG*, and *ABO*, there were also 13 loci reaching genome-wide significance in the meta-analysis. However, these loci did not achieve genome-wide significance in the discovery stage, nor had they been previously identified as PE-associated variants. There was currently insufficient evidence to support the reliability of those association results so we put emphasis on the three loci (*FABP2*, *FGG*, and *ABO*). More East Asian cohorts are needed to verify the associated loci in the future.

PRS has been widely used in the prediction of common diseases, and the PRS model for VTE had been validated in European ancestry. The early genetic risk model of VTE mainly focused on two loci, rs6025 and rs1799963.The Caprini risk assessment model, primarily relying on these two loci, is extensively utilized to predict VTE risk [17]. However, these two variants are almost absent in the EAS population. With the entry into the GWAS era, more loci were used for risk stratification, Crous-Bou et al. established a new risk model based on the 16 SNPs and found that the risk of VTE in patients with high PRS score was 2.02 times that of patients with low PRS score, and achieved better results [55]. Klarin generated a 297 variant polygenic risk score to predict VTE events among patients [45]. Previous research has indicated a reduced accuracy of PRS models when transferred across ancestries [18]. We, therefore, constructed a 288-variant PRS obtained from the EAS population for PE risk prediction. The PRS incorporated population-specific variants and outperformed in EAS population with an AUC of 0.765. Individuals in the top 10% group of PRS had a 5.08-fold of PE risk relative to the general population (30th–70th quantile). However, the model needs further validation in independent datasets with larger sample sizes.

As GWAS have uncovered hundreds of common genetic variants involved in PE susceptibility, our study shed new light on the genetic architecture of PE among Han Chinese population. Nevertheless, like most complex diseases, the common variants discovered in GWAS only explain a fraction of total disease heritability. The rare variants across the whole genome could also play an important role in disease development[56]. Therefore, large-scale sequencing studies of PE in the EAS population are needed to measure the relationship between rare variants and PE risk.

There are several limitations in the current study. Since associations do not imply causation, further research is required to clarify the functional consequences of these novel signals in PE development. We acknowledge the imbalance of sample size and age and sex differences between cases and controls. However, we employed PLINK firth logistic regression with age and sex as covariates in GWAS analysis to control for type-I error issues. Considering the potential risk of inducing biased and spurious associations, we opted not to perform whole genome imputation. Instead, we restricted our imputation to the genomic regions within + / − 500kbp of the *FABP2*, *FGG*, and *ABO* loci, which constituted the main findings of our study. While we acknowledge that this approach may have led to the neglection of genetic signals, we held that these loci exhibited sound reliability. We look forward to expanding our analysis by incorporating more extensive genetic data in future studies.

Nevertheless, our study represents the first multicenter PE genetic study in diverse areas across China, which is a good representative of the Han Chinese population. We revealed through extensive genetic analyses that *FABP2* polymorphism is associated with PE risk and the lipid-metabolic pathways are crucial in the PE development. Although more studies are needed to confirm the value of *FABP2*, the inhibition of *FABP2* is promising to benefit from early intervention in reducing the risk of thrombosis. Our study also demonstrated the utility of applying a population-specific PRS model for PE risk prediction. The clinical use of PRS has the potential to recognize high-risk patients and improve health outcomes through eventual routine implementation as clinical biomarkers.

## Conclusions

In conclusion, this is the first large-scale genetic PE study in EAS. We identified novel risk loci of *FABP2* to expand the global genetic architecture of PE. MR analysis highlighted the importance of lipid-metabolic pathways in PE development. Pharmacological agents modulating blood lipids could be considered in the future for Chinese people to prevent PE. Moreover, we established a population-specific PRS model with improved performance in the EAS population compared to models trained from EUR data. Our study emphasizes the value of investigating diverse ancestral populations in genomic studies, especially for those ethnic groups that are less studied in the global population.

## Supplementary Information

**Additional file 1: Fig. S1.** Regional association plot at genome-wide association study (GWAS) genome-wide significant loci. **Fig. S2.** Principal component analysis (PCA) plot of Han Chinese PE cohort. **Fig. S3.** FUMA Manhattan plot and QQ plot of genome-wide association study (GWAS) meta-analysis. **Fig. S4.** The transfection efficiency of cellular experiments for *FABP2*. **Fig. S5.** Low-density lipoprotein cholesterol (LDL-C) levels of patients with different genotypes of rs1799883. **Fig. S6. **Forest plot for the association of total cholesterol (TC), low-density lipoprotein cholesterol (LDL-C), and triglyceride (TG) with PE. **Fig. S7.** Ancestry-specific polygenic risk score (PRS) ROC plot. **Fig. S8.** Performance of different PRS_VTE_ in the CURES testing set.

**Additional file 2: Table S1.** Independent genome-wide significant lead SNPs associated with pulmonary embolism (PE) in the discovery stage and replication stage. **Table S2.** The allele frequency of identified loci in 1000Genomes. **Table S3.** Replication of associations for the known loci in our cohort. **Table S4.** Association results for genes that were significant in FUMA gene-based analysis. **Table S5.** Polygenic risk score variants. **Table S6.** Polygenic risk score (PRS) quantile and odds ratio (OR).

## Data Availability

For original data, please E-mail the corresponding author.

## Abbreviations

AA: African American
AUC: Area under the curve
DVT: Deep vein thrombosis
EAS: East Asian
EUR: European
GWAS: Genome-wide association study
LD: Linkage disequilibrium
LDL: Low-density lipoprotein
MAF: Minor allele frequency
MR: Mendelian randomization
PCs: Principal components
PE: Pulmonary embolism
PRS: Polygenic risk score
ROC: Receiver operating characteristic curve
SNP: Single nucleotide polymorphism
VTE: Venous thromboembolism
