# Supplementary material for: Genome-wide association analyses identified novel susceptibility loci for pulmonary embolism among Han Chinese population
Source: BMC Med. 2023 Apr 19;21:153. doi: 10.1186/s12916-023-02844-4 (PMC10116678; doi:10.1186/s12916-023-02844-4)
Supplement: Supplementary file 1 — Additional file 1: Fig. S1. Regional association plot at genome-wide association study (GWAS) genome-wide significant loci. Fig. S2. Principal component analysis (PCA) plot of Han Chinese PE cohort. Fig. S3. FUMA Manhattan plot and QQ plot of genome-wide association study (GWAS) meta-analysis. Fig. S4. The transfection efficiency of cellular experiments for FABP2. Fig. S5. Low-density lipoprotein cholesterol (LDL-C) levels of patients with different genotypes of rs1799883. Fig. S6. Forest plot for the association of total cholesterol (TC), low-density lipoprotein cholesterol (LDL-C), and triglyceride (TG) with PE. Fig. S7. Ancestry-specific polygenic risk score (PRS) ROC plot. Fig. S8. Performance of different PRSVTE in the CURES testing set. [file 12916_2023_2844_MOESM1_ESM.docx]

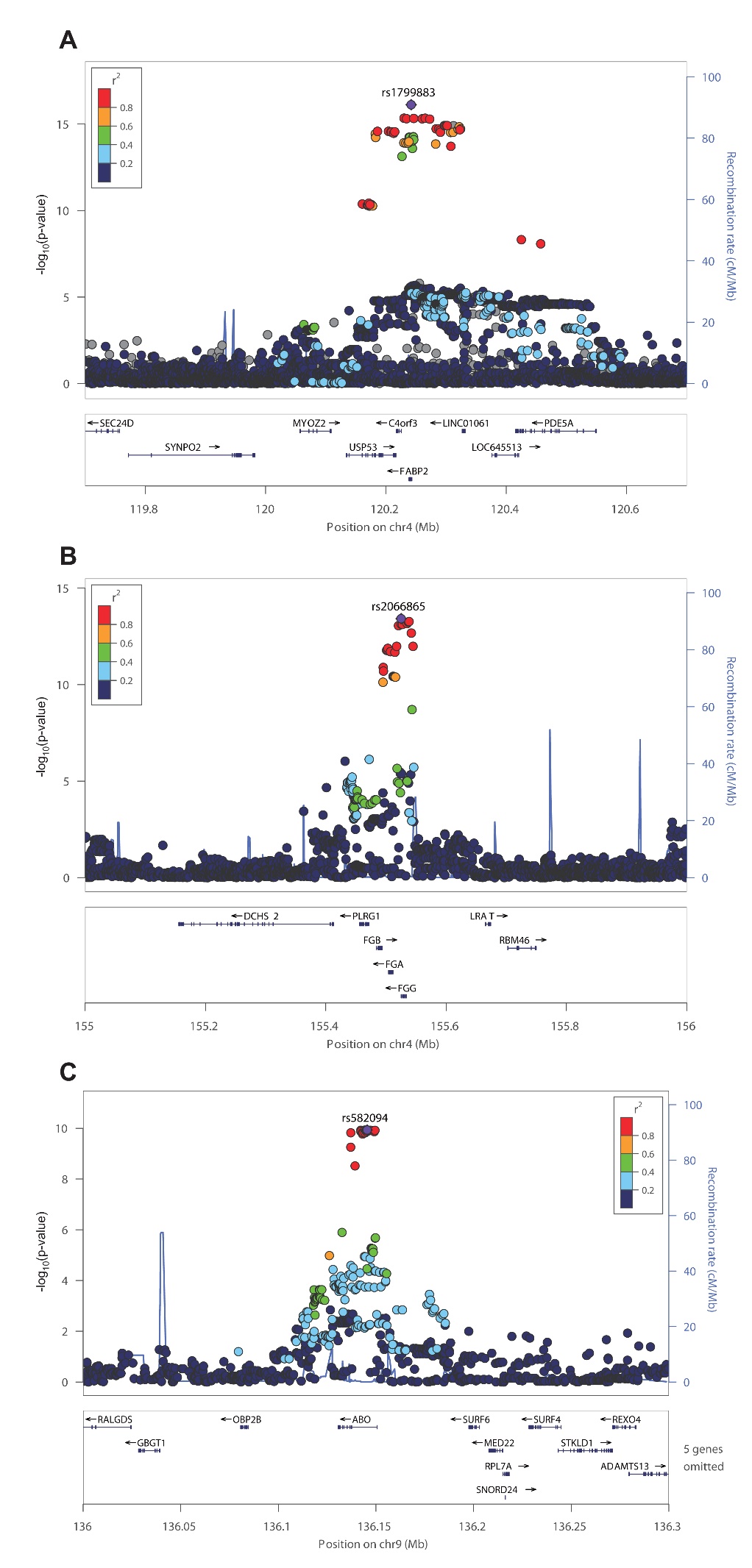


**Fig.S1. Regional association plot at genome-wide association study (GWAS) genome-wide significant loci.** Regional association plots for +/-500kbp regions at the (A) *FABP2*, (B) *FGG*, and (C) *ABO* loci. The plots were produced with Locus Zoom. LD information from the 1000 genome East Asian data was used to calculate the r^2^ between regional variants (colored according to r^2^) and the leading variants (marked with purple diamonds). Recombination rates in the region are visualized as blue curves.**
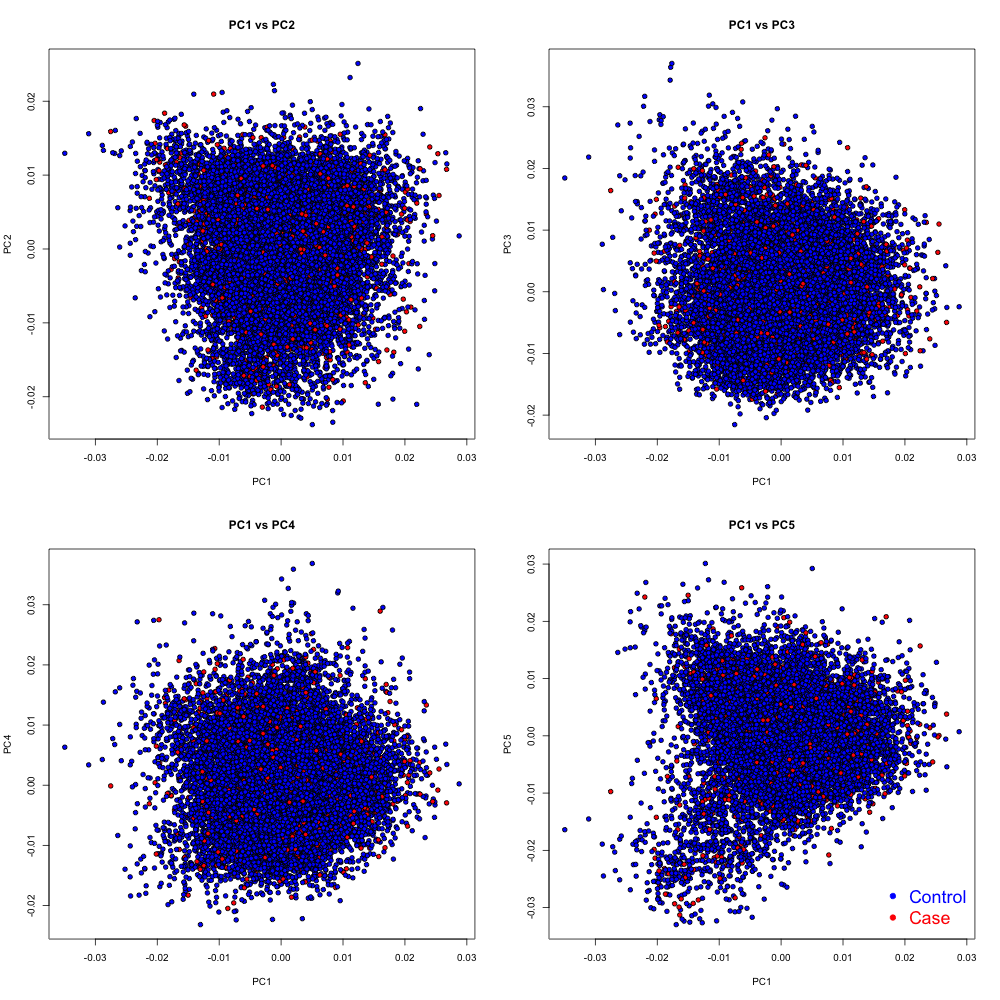
 Fig.S2.** **Principal component analysis (PCA) plot of Han Chinese PE cohort.**

Visualization of principal component analysis (PCA) using the top five PCs obtained from PLINK. (**A**) PC1 and PC2. (**B**) PC1 and PC3. (**C**) PC1 and PC4 and (**D**) PC1 and PC5. The case-control status of each individual is labeled in red and blue, respectively.


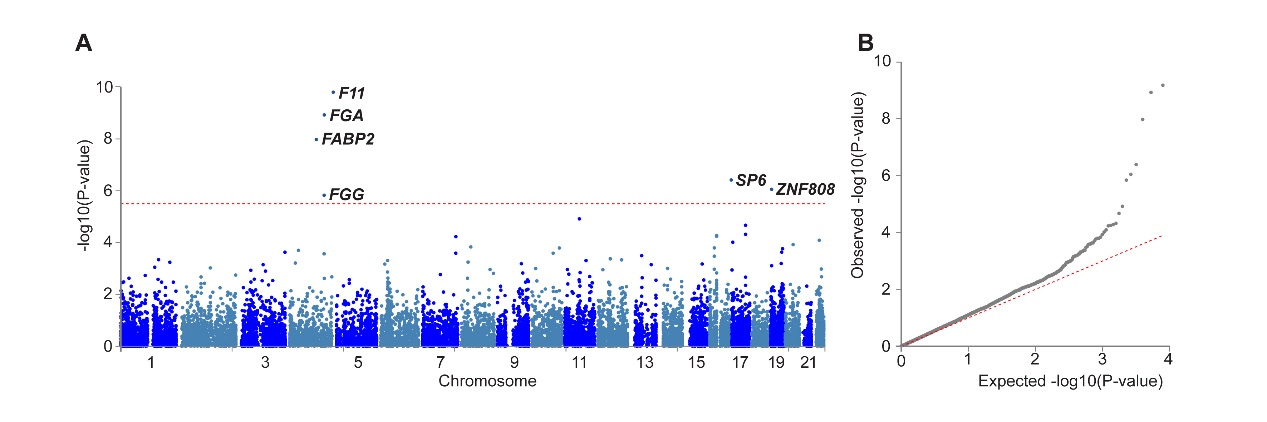


**Fig.S3. FUMA Manhattan plot and QQ plot of genome-wide association study (GWAS) meta-analysis.**

FUMA Gene-based analysis results of the PE association signals. (**A**) Manhattan plot of the results from the gene-based analysis. The y-axis represents gene-level –log_10_(p-values) acquired with FUMA MAGMA. The horizontal red dotted line represents the Bonferroni-corrected threshold after adjusting for 15,756 hypothesis tests. Genes that passed the Bonferroni-corrected threshold are labeled in the figure. (**B**) QQ plot of the gene-based analysis results. The x and y axis represent expected and observed -log_10_(p-values), respectively.

**
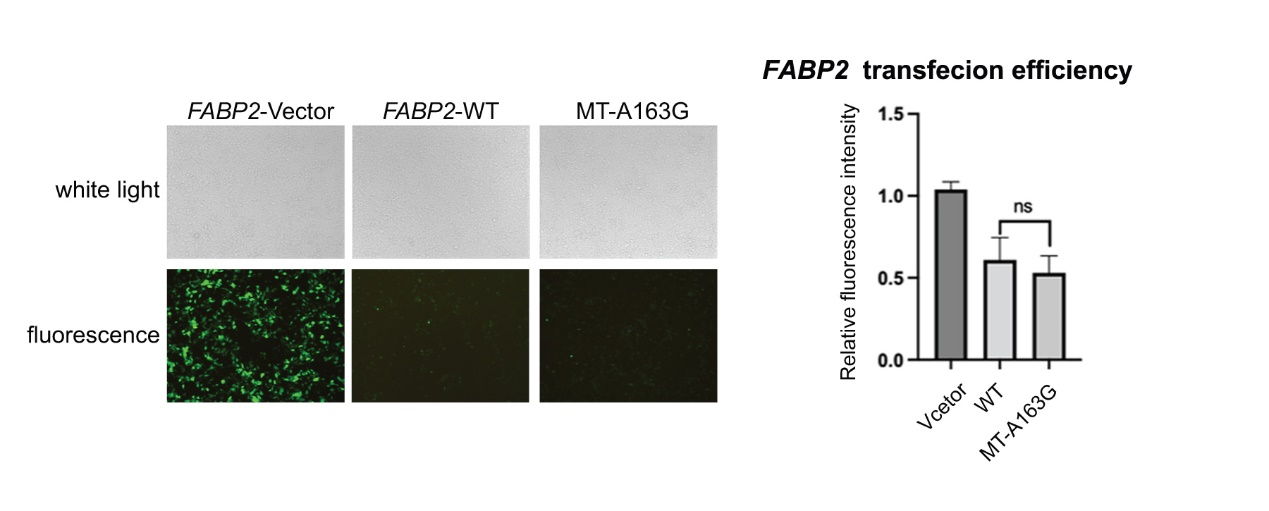
**

**Fig.S4. The transfection efficiency of cellular experiments for *FABP2*.** Transfection efficiency in both *FABP2* (WT/MT-A163G) is lower than Vector in HEK293T, but they are no significant differences for WT VS MT: *FABP2*. ns: not significant.

**
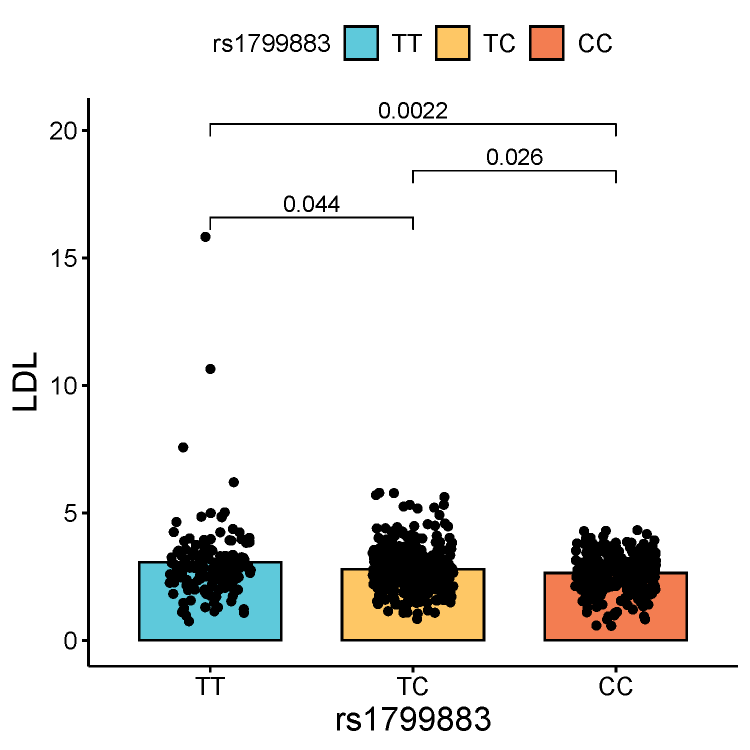
**

**Fig.S5. Low-density lipoprotein cholesterol (LDL-C) levels of patients with different genotypes of rs1799883**. *FABP2* polymorphism is significantly associated with serum LDL-C, persons with TT genotype in rs1799883 tend to have higher LDL than CC genotype.

**
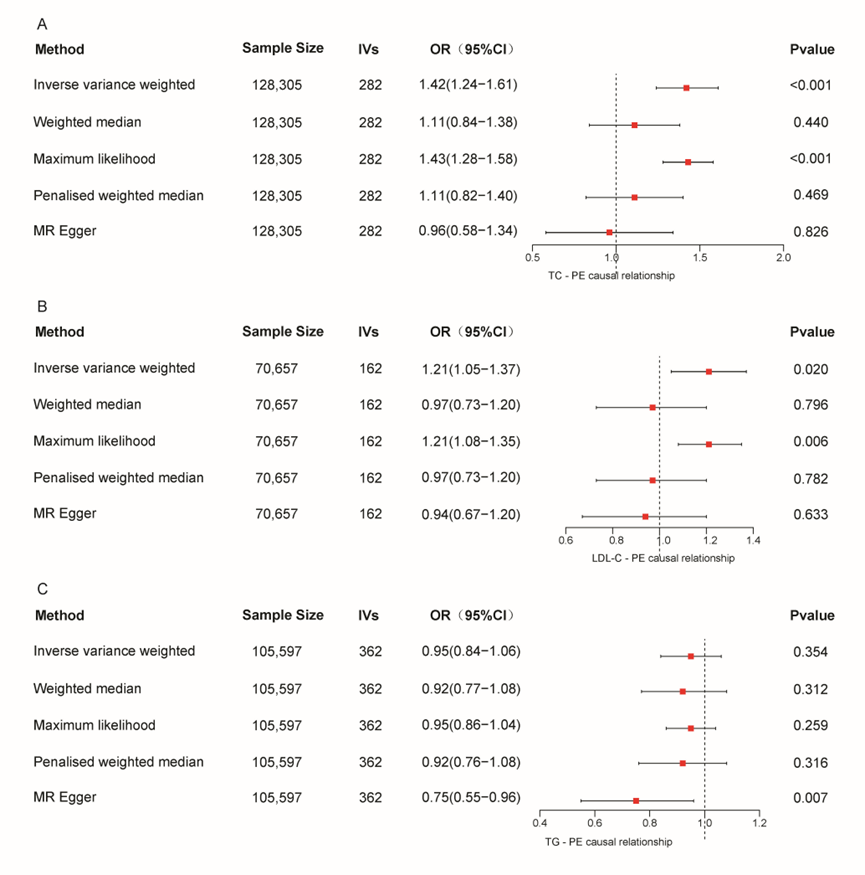
** **Fig.S6. Forest plot for the association of total cholesterol (TC), low-density lipoprotein cholesterol (LDL-C), and triglyceride (TG) with PE.**

Two‐sample Mendelian randomization (MR) was performed in this study. Each box indicates the odds ratio (OR) calculated by each method and horizontal lines represent the 95% confidence interval (CI) of the estimate.

**
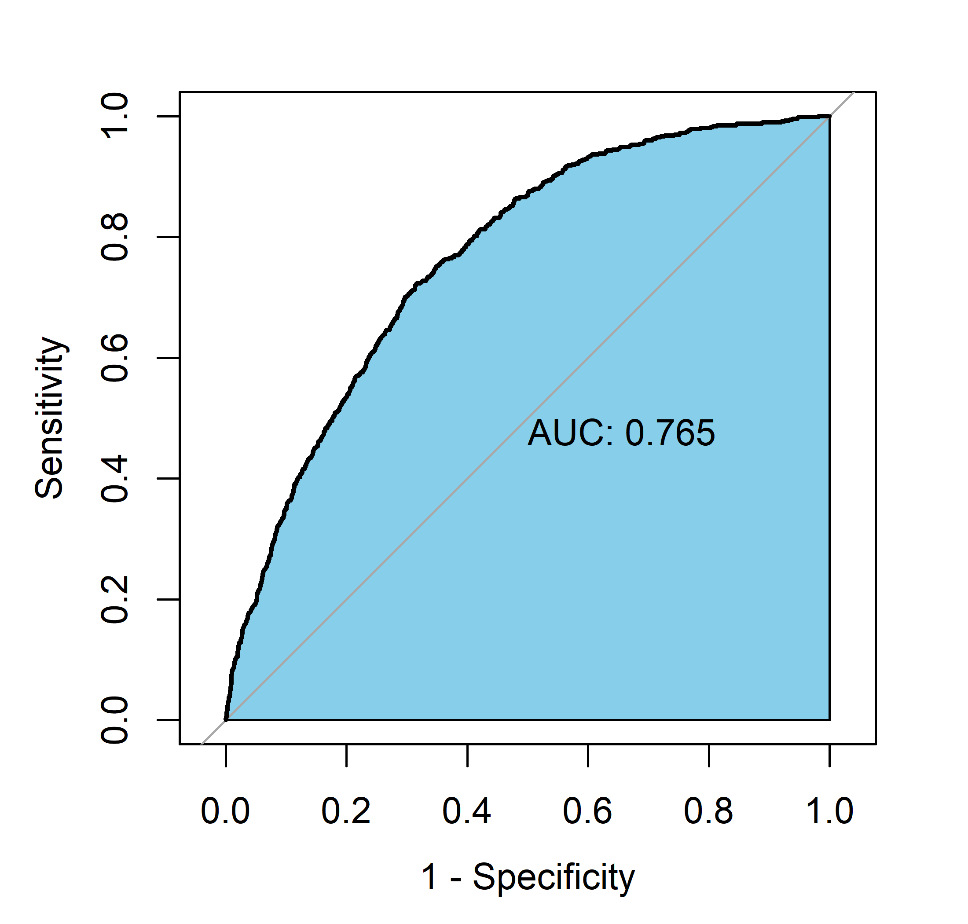
**

**Fig.S7. Ancestry-specific polygenic risk score (PRS) ROC plot**

Receiver operating characteristic curve (ROC) of the constructed PRS model.

**
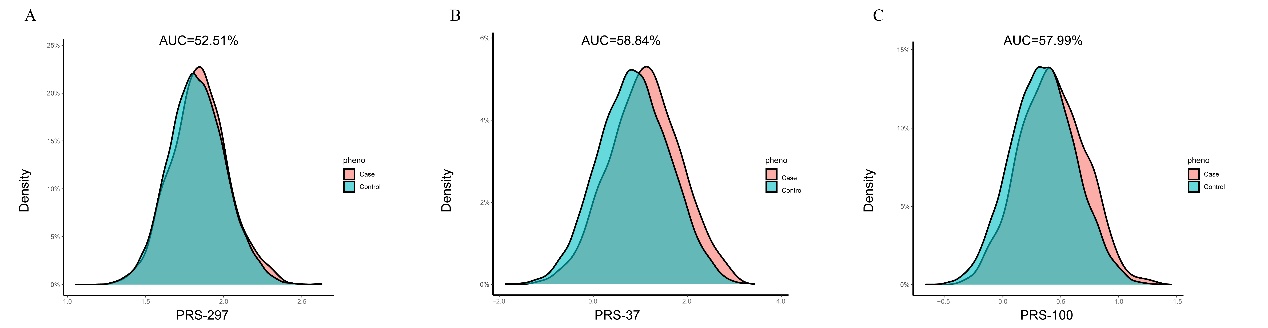
**

**Fig.S8. Performance of different PRS_VTE_ in the CURES testing set.**

Density distribution of (**A**) Khera PRS_2019_ (**B**) Lindstrom PRS_2019_ (**C**) Thibord PRS_2022_. AUC, area under the receiver operating characteristic curve.
