# Supplementary material for: Genome-wide association analyses identified novel susceptibility loci for pulmonary embolism among Han Chinese population
Source: BMC Med. 2023 Apr 19;21:153. doi: 10.1186/s12916-023-02844-4 (PMC10116678; doi:10.1186/s12916-023-02844-4)
Supplement: Supplementary file 2 — Additional file 2: Table S1. Independent genome-wide significant lead SNPs associated with pulmonary embolism (PE) in the discovery stage and replication stage. Table S2. The allele frequency of identified loci in 1000Genomes. Table S3. Replication of associations for the known loci in our cohort. Table S4. Association results for genes that were significant in FUMA gene-based analysis. Table S5. Polygenic risk score variants. Table S6. Polygenic risk score (PRS) quantile and odds ratio (OR). [file 12916_2023_2844_MOESM2_ESM.pdf]

Table S1. Independent genome-wide significant lead SNPs associated with pulmonary embolism (PE) in the discovery stage and replication stage

Chromosome Position	rsID	EA/OA	Nearest Gene	Discovery stage			Replication stage			Meta		
				EAF(case/control)	OR(95%CI)	P-value	EAF(case/control)	OR(95%CI)	P-value	OR(95%CI)	P-value	I^2
4:120241902	rs1799883	T/C	*FABP2*	0.31(0.39/0.31)	1.41(1.25-1.59)	1.05×10^{-8}	0.31(0.39/0.30)	1.43(1.28-1.61)	1.25×10^{-9}	1.42(1.31-1.54)	7.59×10^{-17}	0
4:155525276	rs2066865	A/G	*FGG*	0.51(0.57/0.52)	1.41(1.25-1.58)	9.85×10^{-9}	0.52(0.55/0.52)	1.34(1.20-1.50)	6.19×10^{-7}	1.37(1.26-1.49)	3.81×10^{-14}	0
14:21116815	rs77175619	A/G	*OR6S1*	0.06(0.09/0.06)	1.53(1.25-1.88)	4.28×10^{-5}	0.06(0.10/0.06)	1.82(1.51-2.19)	3.15×10^{-10}	1.68(1.47-1.93)	1.31×10^{-13}	32.71
8:29671268	rs35801946	T/G	*LOC101929470*	0.06(0.02/0.06)	0.33(0.22-0.49)	7.98×10^{-8}	0.06(0.03/0.06)	0.41(0.29-0.59)	1.75×10^{-6}	0.37(0.28-0.49)	9.55×10^{-13}	0
16:88156187	rs4843804	A/G	*ZNF469*	0.05(0.02/0.05)	0.34(0.22-0.51)	4.27×10^{-7}	0.05(0.02/0.06)	0.37(0.25-0.55)	1.02×10^{-6}	0.35(0.26-0.47)	2.12×10^{-12}	0
17:45930539	rs4794202	T/G	*SP6*	0.04(0.01/0.04)	0.19(0.10-0.36)	6.20×10^{-7}	0.04(0.01/0.04)	0.26(0.15-0.45)	1.42×10^{-6}	0.22(0.15-0.34)	5.31×10^{-12}	0
12:130405860	rs708362	T/G	*TMEM132D*	0.05(0.02/0.05)	0.34(0.22-0.52)	4.79×10^{-7}	0.05(0.02/0.06)	0.41(0.28-0.60)	3.63×10^{-6}	0.38(0.29-0.50)	9.94×10^{-12}	0
16:7050441	rs17141995	G/T	*RBFOX1*	0.04(0.01/0.04)	0.23(0.12-0.43)	3.83×10^{-6}	0.03(0.01/0.04)	0.20(0.10-0.39)	2.01×10^{-6}	0.21(0.14-0.34)	3.53×10^{-11}	0
9:136145484	rs582094	T/A	*ABO*	0.43(0.50/0.43)	1.29(1.15-1.45)	2.25×10^{-5}	0.44(0.49/0.43)	1.33(1.19-1.49)	1.18×10^{-6}	1.31(1.21-1.42)	1.16×10^{-10}	0
17:76559574	rs77645935	C/A	*DNAH17*	0.03(0.01/0.03)	0.26(0.14-0.49)	3.12×10^{-5}	0.03(0.01/0.03)	0.14(0.06-0.32)	2.12×10^{-6}	0.21(0.13-0.34)	5.70×10^{-10}	28.67
3:65809842	rs1874320	C/A	*MAGI1*	0.06(0.03/0.06)	0.41(0.28-0.59)	1.93×10^{-6}	0.06(0.03/0.06)	0.54(0.39-0.74)	1.46×10^{-4}	0.48(0.38-0.61)	2.19×10^{-9}	22.38
7:65173823	rs117490078	T/G	*VKORC1L1*	0.10(0.07/0.10)	0.67(0.54-0.84)	6.01×10^{-4}	0.10(0.06/0.11)	0.57(0.45-0.72)	1.92×10^{-6}	0.62(0.53-0.73)	7.78×10^{-9}	10.55
19:53066857	rs12327639	A/G	*ZNF808*	0.07(0.04/0.07)	0.56(0.42-0.75)	1.08×10^{-4}	0.06(0.04/0.07)	0.52(0.38-0.70)	2.56×10^{-5}	0.54(0.44-0.67)	1.12×10^{-8}	0
13:95787496	rs4148515	T/G	*ABCC4*	0.06(0.04/0.06)	0.61(0.45-0.81)	8.93×10^{-4}	0.07(0.03/0.06)	0.49(0.36-0.67)	6.48×10^{-6}	0.55(0.44-0.68)	3.56×10^{-8}	0
13:62785070	rs79753408	T/G	*LINC01075*	0.03(0.01/0.03)	0.22(0.10-0.46)	6.71×10^{-5}	0.03(0.01/0.03)	0.28(0.14-0.54)	1.39×10^{-4}	0.25(0.14-0.41)	3.92×10^{-8}	0
1:213538530	rs7543981	G/A	*RPS6KC1*	0.03(0.02/0.04)	0.43(0.27-0.68)	3.55×10^{-4}	0.03(0.01/0.04)	0.33(0.20-0.55)	2.44×10^{-5}	0.38(0.27-0.54)	4.39×10^{-8}	0

Chromosome Position: Based on hg19/GRCh37 build. rsID: rs number. EA: effect allele. OA: other allele. EAF: effect allele frequency. OR: odds ratio. CI: confidence interval.

Table S2. The allele frequency of identified loci in 1000Genomes

Chromosome Position	rsID	EA/OA	Nearest Gene	Global	African	East Asian	Europe	South Asian	American
4:120241902	rs1799883	T/C	*FABP2*	0.25	0.22	0.25	0.27	0.31	0.23
4:155525276	rs2066865	A/G	*FGG*	0.70	0.65	0.56	0.78	0.76	0.79
14:21116815	rs77175619	A/G	*OR6S1*	0.03	0.00	0.06	0.04	0.01	0.03
8:29671268	rs35801946	T/G	*LOC101929470*	0.16	0.33	0.05	0.14	0.08	0.10
16:88156187	rs4843804	A/G	*ZNF469*	0.13	0.25	0.06	0.05	0.11	0.18
17:45930539	rs4794202	T/G	*SP6*	0.12	0.07	0.05	0.16	0.19	0.16
12:130405860	rs708362	T/G	*TMEM132D*	0.04	0.08	0.04	0.02	0.03	0.01
16:7050441	rs17141995	G/T	*RBFOX1*	0.05	0.07	0.04	0.05	0.02	0.09
9:136145484	rs582094*	T/A	*ABO*	0.37	0.43	0.37	0.36	0.42	0.28
17:76559574	rs77645935	C/A	*DNAH17*	0.06	0.16	0.04	0.00	0.01	0.06
3:65809842	rs1874320	C/A	*MAGI1*	0.31	0.48	0.08	0.42	0.18	0.33
7:65173823	rs117490078	T/G	*VKORC1L1*	0.03	0.00	0.11	0.01	0.01	0.04
19:53066857	rs12327639	A/G	*ZNF808*	0.27	0.43	0.04	0.31	0.18	0.34
13:95787496	rs4148515	T/G	*ABCC4*	0.20	0.41	0.07	0.15	0.12	0.16
13:62785070	rs79753408	T/G	*LINC01075*	0.01	0.00	0.03	0.00	0.00	0.00
1:213538530	rs7543981	G/A	*RPS6KC1*	0.04	0.08	0.03	0.03	0.02	0.03

Chromosome Position: Based on hg19/GRCh37 build. rsID: rs number. EA: effect allele. OA: other allele.

*:data from gnomAD

Table S3. Replication of associations for the known loci in our cohort

Chromosome Position	rsID	Prior	Nearest Gene	Discovery stage		Replication stage		Meta	
				OR (95% CI)	P-value	OR (95% CI)	P-value	OR (95% CI)	P-value
9:136139265	rs657152	Ref. 38.39	*ABO*	1.23(1.10-1.39)	4.39×10^{-4}	1.31(1.17-1.47)	3.00×10^{-6}	1.28(1.18-1.38)	6.83×10^{-9}
9:136149229	rs505922	Ref. 38.39	*ABO*	1.29(1.15-1.45)	2.39×10^{-5}	1.32(1.18-1.48)	1.74×10^{-6}	1.31(1.20-1.42)	3.81×10^{-14}
9:136149722	rs630014	Ref. 38.39	*ABO*	0.82(0.73-0.93)	1.88×10^{-3}	0.80(0.71-0.90)	3.44×10^{-4}	0.81(0.74-0.88)	1.31×10^{-13}
9:136154867	rs495828	Ref. 39	*ABO*	1.13(0.98-1.29)	0.08747	1.27(1.12-1.45)	2.55×10^{-4}	1.09(0.89-1.34)	0.4146
9:136137065	rs687621	Ref. 40	*ABO*	1.25(1.11-1.40)	2.52×10^{-4}	1.33(1.19-1.49)	1.06×10^{-6}	0.80(0.55-1.16)	0.2353
9:136137106	rs687289	Ref. 41	*ABO*	1.27(1.13-1.42)	7.15×10^{-5}	1.33(1.18-1.49)	1.27×10^{-6}	1.30(1.20-1.41)	4.40×10^{-10}
4:155501188	rs6825454	Ref. 39	*FGA*	1.37(1.22-1.54)	1.29×10^{-7}	0.26(0.15-0.45)	1.42×10^{-6}	0.22(0.15-0.34)	5.31×10^{-12}
4:155511897	rs2070011	Ref. 41	*FGA*	0.75(0.67-0.84)	1.86×10^{-6}	0.75(0.66-0.84)	8.42×10^{-7}	0.75(0.69-0.81)	7.17×10^{-12}
4:155525276	rs2066865	Ref. 3.39.42.43	*FGG*	1.41(1.25-1.58)	9.85×10^{-9}	1.34(1.20-1.50)	6.19×10^{-7}	1.37(1.26-1.49)	3.81×10^{-14}
4:155543369	rs6536024	Ref. 40	*FGG*	0.73(0.64-0.82)	6.76×10^{-7}	0.80(0.71-0.90)	2.87×10^{-4}	0.76(0.70-0.83)	1.40×10^{-9}
11:46760756	rs3136516	Ref. 42	*F2*	0.82(0.70-0.96)	0.01383	1.03(0.89-1.19)	0.6869	0.93(0.83-1.03)	0.169
1:169511755	rs4524	Ref. 3	*F5*	0.84 (0.73-0.98)	0.02159	0.90(0.78-1.03)	0.1382	0.87(0.79-0.96)	7.82×10^{-3}
4:187188094	rs4253399	Ref. 40	*F11*	1.21(1.07-1.37)	2.62×10^{-3}	1.30(1.16-1.47)	1.49×10^{-5}	1.26(1.15-1.37)	2.03×10^{-7}
3:93650604	rs6795524	Ref. 44	*PROS1*	1.52(1.12-2.05)	6.53×10^{-3}	1.21(0.89-1.65)	0.2186	1.35(1.09-1.68)	5.07×10^{-3}
20:33777612	rs6087685	Ref. 3	*PROCR*	1.30(1.08-1.55)	4.92×10^{-3}	1.13(0.93-1.36)	0.2146	1.21(1.06-1.38)	3.94×10^{-3}
1:169135127	rs16861990	Ref. 39.41	*NME7*	0.72(0.52-1.00)	0.04731	1.03(0.79-1.35)	0.8149	0.88(0.72-1.08)	0.2825
19:10742170	rs2288904	Ref. 3.44	*SLC44A2*	0.88(0.78-1.00)	0.05002	0.92(0.82-1.04)	0.1729	0.90(0.83-0.98)	0.01932
20:23050806	rs2144940	Ref. 16	*THBD*	1.06(0.82-1.37)	1.08×10^{-4}	1.22(0.95-1.56)	0.1121	1.14(0.95-1.36)	0.1497
6:71623253	rs1304029	Ref. 45	*SMAP1/B3GAT2*	0.98(0.87-1.10)	0.6848	1.02(0.91-1.15)	0.6904	1.00(0.92-1.09)	0.9977
19:33899065	rs731839	Ref. 44	*PEPD*	1.11(0.99-1.25)	0.07309	1.06(0.95-1.19)	0.3029	1.08(1.00-1.18)	0.04665
19:55538980	rs1654425	Ref. 44	*GP6*	0.51(0.27-0.97)	0.03919	1.00(0.62-1.60)	0.9967	0.75(0.52-1.10)	0.2182
3:39188182	rs13084580	Ref. 44	*CSRNP1*	1.23(1.04-1.44)	0.0157	1.22(1.04-1.43)	0.01653	1.22(1.09-1.37)	6.61×10^{-4}

Chromosome Position: Based on hg19/GRCh37 build. rsID: rs number.
OR and P value: odds ratio and P value in the current study. CI: confidence interval.

Table S4. Association results for genes that were significant in FUMA gene-based analysis

Gene	Chromosome Position	Description	P-value
F11	chr4:187,187,099-187,210,835	Coagulation Factor XI	6.72×10^{-10}
FGA	chr4:155,504,278-155,511,918	Fibrinogen Alpha Chain	1.20×10^{-9}
FABP2	chr4:120,238,405-120,243,545	Fatty Acid Binding Protein 2	1.07×10^{-8}
SP6	chr17:45,922,279-45,933,240	Sp6 Transcription Factor	4.12×10^{-7}
ZNF808	chr19:53,030,905-53,067,717	Zinc Finger Protein 808	9.13×10^{-7}
FGG	chr4:155,525,286-155,534,119	Fibrinogen Gamma Chain	1.45×10^{-6}

Chromosome Position: Based on hg19/GRCh37 build.

Table S5. Polygenic risk score variants

Chromosome Position	rsID	EA/OA	P-value	beta
4:155525276	rs2066865	A/G	9.85×10^{-9}	0.34146
4:120241902	rs1799883	T/C	1.05×10^{-8}	0.34359
6:49646509	rs1321615	A/C	7.42×10^{-8}	0.719789
8:29671268	rs35801946	T/G	7.98×10^{-8}	-1.12024
16:88156187	rs4843804	A/G	4.27×10^{-7}	-1.08412
12:130405860	rs708362	T/G	4.79×10^{-7}	-1.07734
17:45930539	rs4794202	T/G	6.2×10^{-7}	-1.68147
3:65809842	rs1874320	C/A	1.93×10^{-6}	-0.89551
14:93049931	rs2402170	C/A	2.37×10^{-6}	-0.31485
13:31696502	rs9315112	T/C	2.94×10^{-6}	0.377751
16:7050441	rs17141995	G/T	3.83×10^{-6}	-1.48192
6:155727925	rs78281438	G/T	5.58×10^{-6}	0.337186
1:115437986	rs6661511	A/G	7.94×10^{-6}	0.502592
2:215635794	rs3768716	C/T	8.02×10^{-6}	0.333611
22:30745204	rs117237368	T/G	8.4×10^{-6}	-0.77914
22:24227246	rs6003939	C/A	9.48×10^{-6}	0.414755
14:88375104	rs393691	T/G	1.02×10^{-5}	0.572673
2:128169899	rs6753288	A/G	1.07×10^{-5}	0.347836
8:42195975	rs2307155	A/C	1.12×10^{-5}	-1.28772
3:16928027	rs114766804	C/T	1.48×10^{-5}	0.443403
15:51684980	rs8032460	C/T	1.73×10^{-5}	0.468753
11:116603677	rs1893460	A/G	2.33×10^{-5}	0.257738
9:136149229	rs505922	C/T	2.39×10^{-5}	0.252314
1:175813319	rs12082852	T/G	2.44×10^{-5}	-1.08058
13:50389401	rs75787368	C/T	2.83×10^{-5}	0.703592
14:95586685	rs117875424	A/G	2.84×10^{-5}	0.655964
20:2166427	rs6082335	T/C	2.95×10^{-5}	0.276874
17:76559574	rs77645935	C/A	3.12×10^{-5}	-1.33522
12:86749652	rs117500272	T/C	3.24×10^{-5}	0.632335
11:11699795	rs12364962	C/T	3.29×10^{-5}	0.492254
2:15930012	rs10929389	C/T	3.57×10^{-5}	-0.29773
16:63461511	rs150740	G/A	3.83×10^{-5}	-0.39943
22:30504207	rs718772	G/A	4.19×10^{-5}	0.330742
7:149445407	rs1622470	G/A	4.28×10^{-5}	0.334327
14:21116815	rs77175619	A/G	4.28×10^{-5}	0.425921
4:114926752	rs78077609	C/T	4.49×10^{-5}	0.599935
10:29370141	rs17760681	T/C	4.62×10^{-5}	0.276115
16:66398525	rs3809648	G/A	4.7×10^{-5}	0.294906
10:90502350	rs376264	G/A	4.73×10^{-5}	-0.25438
2:136879693	rs75238552	A/C	4.81×10^{-5}	-0.91105
11:114186645	rs10891647	C/T	5.01×10^{-5}	0.3257
2:135165813	rs4954144	T/G	5.35×10^{-5}	0.362558
3:58512375	rs1127743	C/T	5.39×10^{-5}	0.271553
1:225375468	rs12729289	T/C	5.43×10^{-5}	0.246079
4:4813322	rs1907991	T/C	5.51×10^{-5}	0.249201
4:98240984	rs182314917	C/T	5.69×10^{-5}	0.860355
13:62917158	rs17061254	G/T	5.75×10^{-5}	0.458058
4:4780454	rs75721280	T/C	5.86×10^{-5}	-0.28768
16:12630422	rs12924885	G/A	6.26×10^{-5}	-0.30246
13:67542554	rs7987478	A/G	6.38×10^{-5}	-2.83651
8:93696238	rs72677411	C/T	6.48×10^{-5}	-1.05326
13:62785070	rs79753408	T/G	6.71×10^{-5}	-1.52602
7:139763419	rs6943771	T/C	6.85×10^{-5}	0.314081
6:69237704	rs118048568	A/G	6.86×10^{-5}	-1.01805
18:67286855	rs8097743	A/G	6.89×10^{-5}	0.241376
5:37087250	rs76653927	G/A	6.96×10^{-5}	0.639746
1:9339467	rs9442580	T/C	7.22×10^{-5}	0.587787

8:63237280	rs13260909	C/T	7.55×10^{-5}	0.24059
3:141275436	rs16851483	T/G	7.74×10^{-5}	0.256965
15:37383688	rs2701524	C/T	8.15×10^{-5}	0.245296
3:181502982	rs17540800	T/C	8.44×10^{-5}	0.521172
3:25706277	rs10433573	C/A	8.92×10^{-5}	-0.28489
8:17300360	rs140242583	T/C	8.97×10^{-5}	0.678034
9:17875698	rs79627944	G/A	9.31×10^{-5}	0.652846
14:36725611	rs77223023	G/T	9.82×10^{-5}	0.427879
3:12916106	rs6808492	C/T	0.000103	0.590006
12:83808150	rs76144234	G/T	0.000107	-1.24584
19:53066857	rs12327639	A/G	0.000108	-0.58304
1:69685100	rs4650137	C/T	0.00011	0.242946
6:72530409	rs12525765	C/A	0.000112	0.528273
16:7946087	rs73502833	A/G	0.000123	0.328584
9:31236422	rs1360665	C/A	0.000125	0.366724
3:43156535	rs76876334	T/G	0.000125	0.33146
16:11016593	rs116882824	T/G	0.000127	-0.94649
2:237678052	rs719649	C/T	0.00013	0.330023
12:94959873	rs79368348	T/C	0.000131	0.812263
2:219553468	rs16859180	T/C	0.000135	0.37363
4:5711368	rs3774854	G/A	0.000138	0.227136
10:34323378	rs1705013	G/A	0.00014	-0.26605
11:115035485	rs7131142	C/T	0.000143	-0.24501
10:85568009	rs10887157	A/C	0.000144	0.479335
19:7411780	rs6603095	G/A	0.000145	0.300105
4:39457430	rs2687956	T/C	0.000149	0.247641
4:180517056	rs68158635	T/C	0.000149	-0.27998
3:147866462	rs67416703	T/C	0.00015	-0.47321
15:47509599	rs8040414	A/C	0.00015	0.372253
3:114530679	rs9289004	G/A	0.000154	0.53004
12:106672618	rs7308143	A/G	0.000157	0.298622
22:47530307	rs144351340	A/G	0.000162	0.551584
15:31665443	rs4779520	C/T	0.000163	-0.24692
11:133170270	rs2186826	C/A	0.000163	-0.35753
19:3171565	rs10421389	G/A	0.000165	-0.32117
13:35252882	rs9572094	T/C	0.000168	0.223943
6:109049607	rs6910666	G/A	0.00017	-0.22377
5:42745904	rs190782568	A/G	0.000171	0.235862
9:73574860	rs945688	T/C	0.000179	-0.22302
16:81905504	rs12447917	G/A	0.000181	0.222343
10:116347162	rs2483579	A/G	0.000188	-0.22652
20:56776961	rs6123776	C/T	0.000188	-0.278
2:100145818	rs116854723	G/T	0.0002	-0.71009
4:158727739	rs1448439	G/T	0.000206	0.34288
13:52318012	rs150363035	G/A	0.000206	-1.33067
21:46960895	rs13050920	C/T	0.000206	-0.22289
18:62526264	rs138232306	G/A	0.000211	0.410121
15:88060128	rs372071390	T/G	0.000211	-1.26054
17:78992409	rs7225916	G/T	0.000214	0.219938
4:131018512	rs71613297	G/A	0.000214	0.226338
6:1606384	rs62390610	T/G	0.000221	-1.13227
1:21757665	rs114453875	C/T	0.000224	-1.41223
4:132948418	rs6812658	G/A	0.000227	-0.3169
6:143058357	rs17071973	T/C	0.000229	-0.35639
5:37909501	rs62360405	A/C	0.000231	-0.32739
8:106573578	rs3735953	C/T	0.000234	0.217528
8:64198963	rs57808486	G/A	0.000235	0.239804
7:150526345	rs76036237	G/T	0.000236	0.368801

15:34158266	rs1044129	G/A	0.000244	-0.22377
21:38308911	rs1065758	A/G	0.000249	0.429182
2:237811515	rs4663236	A/G	0.000255	0.241376
2:181803749	rs10210356	G/A	0.000258	-0.62923
19:32483186	rs17717716	C/T	0.000261	0.217528
14:53151109	rs7150290	C/T	0.000271	0.241376
6:164647755	rs9459032	C/T	0.000272	0.216723
6:67132658	rs2670358	C/A	0.000273	0.214305
12:119362496	rs79902143	T/C	0.000273	-0.56793
13:106202606	rs77481115	A/G	0.000276	0.785726
8:70834061	rs56299958	T/C	0.000276	-0.75099
1:230687711	rs853452	T/C	0.000278	0.219136
18:2082393	rs532950	T/C	0.00028	0.244514
10:21311248	rs10828176	T/G	0.000281	0.215918
4:38760100	rs77778664	T/C	0.000281	0.411447
14:22241710	rs11157216	T/C	0.000282	0.251537
17:69794912	rs11654836	C/T	0.000282	-0.61897
12:48399612	rs1859443	A/G	0.000285	0.264669
2:42517516	rs7569544	A/C	0.000291	0.246079
7:147918464	rs6947963	T/G	0.000293	-0.25644
15:73274669	rs28424084	G/A	0.000296	0.214305
17:18077175	rs854800	T/C	0.000296	0.219136
5:24456465	rs59451037	A/C	0.000298	-0.72093
6:97123214	rs76503183	T/G	0.000299	-1.22248
2:218174378	rs116547900	T/C	0.000299	-0.85402
11:17835042	rs1548528	A/C	0.000302	0.279146
16:21729214	rs215902	T/C	0.000303	-0.21716
11:17861074	rs59143224	A/G	0.000306	0.3257
8:142730519	rs10111342	T/C	0.000309	-0.22803
1:210836847	rs1890843	C/T	0.000309	0.263902
16:1151323	rs197075	A/G	0.000311	0.263902
22:28025519	rs117623535	G/A	0.000311	-0.50999
6:116032524	rs961319	G/A	0.000312	0.552159
20:30643595	rs6121328	T/C	0.000315	0.765933
9:24985660	rs1461340	A/G	0.000315	0.225541
8:143599594	rs75573695	T/C	0.000322	0.720762
11:44723896	rs140972488	T/G	0.000324	-1.2888
7:50436476	rs117963681	T/C	0.000327	0.328584
2:67935402	rs12465679	G/A	0.000334	0.214305
16:63460173	rs76843065	G/A	0.000342	0.241376
1:95068347	rs1772895	A/G	0.00035	-0.24067
8:63817743	rs16929812	G/A	0.00035	-0.31759
14:40975244	rs2150522	G/A	0.000353	0.227136
1:213538530	rs7543981	G/A	0.000355	-0.8391
9:137777132	rs12684476	A/G	0.000358	-1.20564
6:91783441	rs16883972	G/A	0.000361	0.297137
3:121759644	rs6762289	C/A	0.000362	-0.67315
7:14423100	rs7785682	G/A	0.000366	-0.28942
19:36583234	rs7250473	T/G	0.000369	0.611395
9:11073873	rs10959509	C/T	0.000371	-0.26253
10:13534851	rs2251555	G/A	0.000376	0.220741
5:31059476	rs13167643	A/C	0.000377	-0.32642
10:118522665	rs1665672	G/T	0.000377	0.230318
16:65801698	rs1966503	A/G	0.000379	0.599386
6:57127760	rs9475804	A/G	0.000384	-0.31677
19:52836147	rs55952017	T/C	0.000385	0.630207
10:72991385	rs7893421	T/C	0.000395	0.21188
6:93494267	rs9452114	A/C	0.000398	-1.60147

2:68515530	rs6740317	G/A	0.000409	0.20945
6:34439749	rs9469791	A/G	0.000409	0.220741
6:136099892	rs12198880	C/A	0.00041	-0.65489
8:128428581	rs188813106	T/G	0.00041	-1.20331
15:59855226	rs12902622	T/C	0.000413	0.211071
8:25816376	rs4872381	T/C	0.000416	0.291923
5:178748645	rs456356	T/C	0.000417	0.303801
4:20100285	rs4697140	C/A	0.000419	-0.57554
4:113779891	rs1448221	G/T	0.000424	0.261595
8:17419299	rs2705062	A/G	0.000424	0.221542
9:92853543	rs12115490	T/C	0.000425	0.263133
3:42368008	rs11715412	G/A	0.00043	0.234281
5:104596665	rs2431976	C/T	0.000431	0.324255
19:42053554	rs758768	T/C	0.000431	0.658556
4:172448151	rs11728304	A/G	0.000435	0.207014
5:57787376	rs2307117	G/A	0.000437	0.253867
1:182168885	rs1689800	G/A	0.000441	0.216723
11:71016690	rs190623923	T/C	0.000447	0.615186
12:125608492	rs1080910	C/T	0.000447	-0.21084
14:81192479	rs2619676	G/A	0.00045	-0.23636
14:70883029	rs375840470	C/A	0.000455	-1.58523
13:107801656	rs147567900	A/G	0.000455	0.652846
19:29080584	rs929440	T/C	0.000458	0.208639
1:220319007	rs75519000	G/A	0.000463	-0.52138
16:14430532	rs2867417	A/C	0.000464	0.207014
3:157675069	rs9290009	A/G	0.000465	-0.32076
1:162062596	rs12069128	T/G	0.000468	0.251537
14:66242314	rs10132765	G/A	0.000468	-0.23813
9:7780039	rs10976571	T/C	0.000471	0.280657
12:46222529	rs147424196	G/A	0.000473	0.441476
5:134824264	rs17168440	T/G	0.000474	-0.24731
5:40859489	rs77581485	A/C	0.000474	0.215918
2:10577645	rs74457099	T/C	0.000476	0.51641
20:56705287	rs6015200	C/T	0.000481	0.212689
9:11018077	rs12346562	A/C	0.000481	-0.65991
3:170359696	rs189032268	C/A	0.000483	-1.57697
14:39426108	rs1022713	G/A	0.000486	-0.21183
4:63401079	rs6826037	A/G	0.000489	0.248421
12:129394746	rs11060010	A/G	0.000491	-0.27892
4:25085481	rs16876768	T/C	0.000492	0.366724
16:54493354	rs55654587	A/G	0.000497	-0.36169
6:54086108	rs3957360	C/T	0.000501	0.532391
12:94126925	rs10859567	T/G	0.000504	-0.21109
16:82203758	rs2303262	C/T	0.000505	-0.29397
21:47362573	rs439180	A/G	0.000507	0.263133
12:71879943	rs12297272	T/G	0.000507	0.521172
18:57388538	rs10163697	A/G	0.000508	0.242946
13:110404191	rs117415218	C/T	0.000512	0.515813
19:19293192	rs2238671	T/C	0.000512	0.319181
1:11862214	rs9651118	C/T	0.000515	-0.23307
13:100579166	rs7324649	G/A	0.000523	-0.2189
8:70859624	rs7836791	C/T	0.000524	-0.25877
1:221819994	rs7525746	T/C	0.000524	0.217528
12:93732312	rs11106986	C/A	0.000527	0.716375
7:66811313	rs7804537	A/G	0.000529	-0.26318
7:157195623	rs118124567	T/C	0.000531	-0.492
3:112469992	rs820554	C/T	0.000541	0.231905
3:93842702	rs13094371	G/A	0.000542	0.559616

2:239103574	rs35393292	A/G	0.000545	0.24373
2:68886057	rs6735917	A/G	0.000549	-0.22164
8:15623609	rs34145513	T/C	0.000556	0.502592
8:98394419	rs2853292	G/A	0.000556	0.207014
19:3872565	rs35288732	T/C	0.000561	0.206201
14:77357411	rs140059140	A/C	0.000563	-0.59167
8:106231172	rs1350723	A/G	0.00057	-0.20973
8:73821889	rs1482019	A/G	0.00057	-0.31718
1:63231401	rs11807368	G/T	0.000571	-0.21791
5:76455453	rs10044684	A/G	0.000572	-0.23965
6:156972117	rs1926930	A/G	0.000576	0.583332
4:137539102	rs62309592	G/A	0.000576	-0.21208
9:120114228	rs10818029	C/T	0.000582	-0.48809
4:77392092	rs6843293	G/A	0.000585	0.212689
11:12596007	rs11022423	C/A	0.000586	-1.05527
17:13643413	rs78102228	A/G	0.000587	-0.65952
19:29414631	rs4805303	T/C	0.00059	0.202941
9:73615652	rs138455065	G/A	0.00059	0.427879
18:53918924	rs2538025	C/T	0.000591	0.383219
12:123837291	rs77866843	A/C	0.000591	0.43761
18:29424289	rs9966530	A/G	0.000592	-0.20702
5:65300102	rs74290406	T/C	0.000593	0.476234
22:37462926	rs2235321	A/G	0.000595	-0.20825
3:43428108	rs145068762	T/C	0.000599	0.448525
2:115196802	rs72949839	G/A	0.000601	-0.31993
7:65173823	rs117490078	T/G	0.000601	-0.39319
3:158628954	rs75245476	A/G	0.000602	0.567017
4:182142593	rs10002947	G/T	0.000604	0.460584
7:83715504	rs2527035	C/T	0.000604	0.290428
10:112543615	rs2146545	C/T	0.00061	0.241376
9:3801316	rs2124210	A/C	0.000611	0.254642
12:70101900	rs118138993	A/G	0.000613	0.627007
11:69665620	rs7481423	C/A	0.000616	-0.60039
19:50492291	rs6509451	A/G	0.000617	0.215111
6:38779140	rs1738225	C/T	0.000617	0.202941
13:63946948	rs75416701	T/C	0.000624	0.374318
13:47341682	rs9595530	A/G	0.000625	-0.27391
9:2227217	rs7024071	T/G	0.000628	0.220741
7:150827104	rs78061884	A/G	0.000629	0.408128
4:155976058	rs72967365	G/T	0.000633	0.290428
3:19972827	rs71316238	A/G	0.000645	-0.96233
4:43879438	rs77179346	A/G	0.000654	0.337186
20:59605562	rs6101198	C/A	0.000656	-0.2101
4:187205929	rs4253425	T/C	0.000657	-0.47177
12:130003842	rs3853782	A/G	0.000659	0.304539
15:42271067	rs1614300	G/T	0.000666	0.200489
2:2227894	rs12052817	A/C	0.000669	-2.41631
20:18633244	rs77487090	T/G	0.000671	-1.15233
7:155904974	rs73482924	A/C	0.000673	-1.30748
2:133040424	rs4954289	C/T	0.000678	-0.34588
18:74308179	rs77791992	G/A	0.000678	0.588897
11:788007	rs6597982	G/A	0.000683	0.208639
14:101224922	rs2400940	G/A	0.000688	-0.26514
1:30885210	rs650676	C/T	0.00069	0.228728
1:243332619	rs12039211	A/G	0.000696	-0.5704
2:241333141	rs1317565	T/C	0.000697	-0.35041
8:104187968	rs954782	A/G	0.000697	0.19967

Table S6. Polygenic risk score (PRS) quantile and odds ratio (OR)

**Quantile**	**OR**	**95%CI**
< 10th	0.14	0.065-0.296
10-20th	0.12	0.053-0.268
20-30th	0.36	0.222-0.588
30-70th	1	1
70-80th	2.15	1.666-2.772
80-90th	2.73	2.149-3.470
< 90th	5.08	4.109-6.282

Estimate of OR and 95% CI when taking the 30-70th quantile of inviduals as reference group.OR: odds ratio. CI: confidence interval
